# Geospatial variation in co‐occurrence networks of nitrifying microbial guilds

**DOI:** 10.1111/mec.14893

**Published:** 2018-11-03

**Authors:** Christopher M. Jones, Sara Hallin

**Affiliations:** ^1^ Swedish University of Agricultural Sciences Department of Forest Mycology and Plant Pathology Uppsala Sweden

**Keywords:** ammonia‐oxidizing communities, network analysis, nitrite‐oxidizing communities, soil microbiology, spatial mapping

## Abstract

Microbial communities transform nitrogen (N) compounds, thereby regulating the availability of N in soil. The N cycle is defined by interacting microbial functional groups, as inorganic N‐products formed in one process are the substrate in one or several other processes. The nitrification pathway is often a two‐step process in which bacterial or archaeal communities oxidize ammonia to nitrite, and bacterial communities further oxidize nitrite to nitrate. Little is known about the significance of interactions between ammonia‐oxidizing bacteria (AOB) and archaea (AOA) and nitrite‐oxidizing bacterial communities (NOB) in determining the spatial variation of overall nitrifier community structure. We hypothesize that nonrandom associations exist between different AO and NOB lineages that, along with edaphic factors, shape field‐scale spatial patterns of nitrifying communities. To address this, we sequenced and quantified the abundance of AOA, AOB, and *Nitrospira* and *Nitrobacter* NOB communities across a 44‐hectare site with agricultural fields. The abundance of *Nitrobacter* communities was significantly associated only with AOB abundance, while that of *Nitrospira* was correlated to AOA. Network analysis and geostatistical modelling revealed distinct modules of co‐occurring AO and NOB groups occupying disparate areas, with each module dominated by different lineages and associated with different edaphic factors. Local communities were characterized by a high proportion of module‐connecting versus module‐hub nodes, indicating that nitrifier assemblages in these soils are shaped by fluctuating conditions. Overall, our results demonstrate the utility of network analysis in accounting for potential biotic interactions that define the niche space of nitrifying communities at scales compatible to soil management.

## 1 INTRODUCTION

The nitrogen (N) cycle is best described as a complex network of N‐transformation reactions, driven by a diverse assemblage of different microbial groups (Kuypers, Marchant, & Kartal, 2018). It is the interaction of these groups, through the consumption and production of different forms of inorganic N, which ultimately determines whether N is retained or lost from an ecosystem and if reactive intermediates that could be detrimental for the environment accumulate. One of several pathways that can be either complete (Daims et al., 2015; van Kessel et al., 2015) or shared between different microorganisms is nitrification, in which ammonia (NH_3_) is oxidized into nitrate (NO_3_^−^). This process is of special interest in agricultural soil as NO_3_^−^ is rapidly lost by leaching or by feeding into denitrification, which in turn results in emission of gaseous N compounds. When the pathway is shared, communities of ammonia‐oxidizing archaea (AOA) and bacteria (AOB) produce nitrite (NO_2_^−^), followed by oxidation of NO_2_^−^ to NO_3_^−^ by communities of nitrite‐oxidizing bacteria (NOB). Recent work has shown that the interaction between ammonia and nitrite oxidizers is more complex than this simple mutualistic relationship (Daims, Lücker, & Wagner, 2016). The complexity of interactions between AOA, AOB and NOB communities is exemplified by work demonstrating that certain NOBs are capable of hydrolysing urea to produce NH_3_, resulting in a “reciprocal‐feeding” where ureolytic NOB provides ammonia to urease‐negative AOB that, in turn, produce the NO_2_^−^ that is consumed by NOB (Koch et al., 2015). Also, iron uptake may be facilitated by interaction between AO and NOB communities by complementary sets of siderophore biosynthesis and receptor genes between the two groups (Daims et al., 2016).

The observed interactions suggest that the distribution of nitrifier communities in soil could be determined by associations among specific lineages of AO and NOB. While a large body of work on AO communities in soils exists (Beeckman, Motte, & Beeckman, 2018; Li, Chapman, Nicol, & Yao, 2018; Offre, Spang, & Schleper, 2013; Prosser & Nicol, 2012), fewer studies have examined the NOB communities and even less the interaction or association among different groups of AO and NOB. The genera *Nitrospira* and *Nitrobacter*, within the Nitrospira and α‐proteobacteria, respectively, seem to be the most prevalent NOB in soil ecosystems (Daims et al., 2016; Ke, Angel, Lu, & Conrad, 2013; Li et al., 2018). Niche differentiation has been demonstrated among lineages within each of the four functional groups—AOB, AOA, *Nitrospira* NOB and *Nitrobacter* NOB (Gubry‐Rangin et al., 2011; Maixner, Wagner, & Lücker, 2008; Nowka, Daims, & Spieck, 2015; Wessén et al., 2011; Yao et al., 2013)—yet how the diversity and structure of nitrifying communities are shaped by interactions and other ecological processes that are underlying the co‐occurrence of AO and NOB lineages remains unclear.

The focus of this study was to investigate the spatial distribution of co‐occurring AO and NOB communities and identify edaphic factors associated with unique assemblages of AO and NOB lineages. Wessén et al. (2011) demonstrated that shifts in the abundance and structure of AOA and AOB communities exhibited significant spatial dependence across a 44‐ha farm, and we build from this previous study by also quantifying the abundance of *Nitrospira* and *Nitrobacter* NOB communities as well as determining the diversity and composition of the AOB, AOA, *Nitrospira* NOB and *Nitrobacter* NOB communities by sequencing of functional marker genes specific to each of the four groups. We hypothesized that AO and NOB communities exhibit nonrandom patterns of co‐occurrence across the site. Differences in substrate affinities between AOA and AOB as well as between *Nitrobacter* and *Nitrospira* NOB have been proposed (Nowka et al., 2015; Prosser & Nicol, 2012), although recent studies examining enrichments or pure cultures of these four groups have shown that lineages within each group may have very different physiologies, particularly in regard to optimal or tolerated substrate concentrations (Lehtovirta‐Morley et al., 2016; Sauder et al., 2017). Thus, deterministic processes, including biotic interactions, influencing the assembly of the different functional communities would give rise to a more assortative association of various lineages of AO and NOB. This has been explored in bacterial communities (Barberán, Bates, Casamayor, & Fierer, 2012), but not for interacting functional communities. Significant patterns of spatial co‐occurrence are often cited as indicating biotic interactions being a key driver of community assembly (Diamond, 1975; Gotelli & McCabe, 2002). However, other deterministic processes of community assembly, such as habitat filtering (Horner‐Devine et al., 2007), may also result in nonrandom AO and NOB associations. The use of null models in combination with co‐occurrence metrics allows us to infer whether species pairs are spatially aggregated, segregated or randomly distributed, but they cannot by themselves offer deeper insight on the key processes shaping the observed community without additional information (Blois et al., 2014). We therefore examined co‐occurrence patterns in a spatially explicit context by combining network analyses and community detection with geostatistical mapping to assess whether specific lineages of AO and NOB segregate into distinct subcommunities—or modules—of nitrifying communities and examined the degree to which modules overlapped in terms of spatial distribution and environmental preference across the field site.

## 2 MATERIALS AND METHODS

### 2.1 Field site, soil sampling and soil characterization

The Logården research farm is located in southwest Sweden (58 20′N, 12 38′E) and consists of a 26‐ha area under integrated farm management, and an adjacent 18‐ha area to the north managed according to the Swedish criterion for organic farming (http://www.krav.se) since 1991. Each area is divided into seven fields under a 7‐year crop rotation that includes green manure leys with red clover mixed with grasses or faba beans (Stenberg et al., 2012). Fields under integrated management are tilled by tine cultivation to 10 cm and receive optimal mineral fertilizer inputs, whereas soil in the organic farming system is tilled to a depth of 20 cm using a mouldboard plough, and addition of N is solely through symbiotic N‐fixation in leguminous crops. A total of 51 fixed sampling locations (Supporting information Figure S1) were established throughout the field site based on prior identification of environmental gradients in the field (Söderström & Lindén, 2004), and soil samples were collected in April 2007 by taking 12 soils cores (20 mm diameter, 10 cm depth) at each location and pooling into a single composite sample, followed by passage through a 4‐mm sieve. Soil to be used for molecular analysis was immediately stored at −20°C, and an extensive survey of soil physical and chemical properties for each location was performed. Complete descriptions of analyses of chemical and physical soil parameters are provided in Enwall, Throbäck, Stenberg, Söderström, and Hallin (2010).

### 2.2 DNA extraction and real‐time PCR quantification

Extraction and quantification of soil DNA, and tests for the presence of PCR inhibitors, as well as quantification of AOA and AOB *amoA* genes, were reported previously in Wessén et al. (2011). The *Nitrospira* and *Nitrobacter* NOB were quantified by targeting the *nxrB* gene encoding the nitrite oxidoreductase. *Nitrospira nxrB* genes were amplified using primers nxrB169f and nxrB638r (Pester et al., 2013) generating a 492‐bp fragment, whereas *Nitrobacter nxrB* genes were amplified using primers nxrB1‐F and nxrB1‐R (Vanparys et al., 2007), resulting in a 380‐bp fragment. All reactions were performed in 20 μl volumes containing either 0.5 or 0.3 μM of group‐specific primers for *Nitrospira* and *Nitrobacter*, respectively, 1X iQ SYBR Green Supermix (Bio‐Rad, Hercules CA, USA), 0.5 μg T4 gene 32 product (New England Biolabs, Ipswich MA, USA), and 10 ng soil DNA. Standard curves consisted of 10‐fold serial dilutions of linearized plasmids containing *nxrB* from *Nitrospira delfluvii* or *Nitrobacter winogradsky*, and assays were performed in duplicate for each gene target in a CFX96 Real‐Time PCR detection system (Bio‐Rad). Melt curve analysis was performed at the end of qPCR runs at 72–98°C in 0.5°C increments, and all products were inspected by agarose gel electrophoresis to ensure products were the correct size. Amplification efficiency for the *nxrB* genes ranged from 92% to 96%, with an *r* ^2^ ≥ 0.99 for each gene. Nonspecific amplification was absent in non‐template and negative controls with sterile water. All primers and cycling conditions are provided in Supporting information Table S1. Due to detection of >10% nonspecific sequences of equal size to the target gene in the *Nitrospira nxrB* sequence data set, the total abundance of *Nitrospira nxrB* was corrected for each sample based on the proportion of target reads observed in the sequence data (Supporting information Table S2). There was no need to correct the abundances of the other genes.

### 2.3 Sequencing and bioinformatic analyses of *amoA* and *nxrB* genes

Prior to sequencing and analyses, ARB databases with reference alignments and phylogenies for each gene were generated from sequences obtained from previous studies (AOA *amoA*, Pester et al., 2012; AOB *amoA*, Purkhold, Wagner, Timmermann, Pommerening‐Röser, & Koops, 2003; Mintie, Heichen, Cromack, Myrold, & Bottomley, 2003; *Nitrospira nxrB*, Pester et al., 2013; Daims et al., 2015; van Kessel et al., 2015; *Nitrobacter nxrB*, Vanparys et al., 2007), as well as from metagenome projects deposited in the Integrated Microbial Genome (IMG) database (Markowitz et al., 2012). A full description of alignment and phylogenetic methods used to construct reference databases is provided in Appendix S1: Supplemental Materials and Methods.

The *amoA* genes from AOA and AOB communities as well as the *nxrB* genes from *Nitrospira* and *Nitrobacter* nitrite‐oxidizing communities were amplified from all samples using group‐specific primers for each gene (Supporting information Table S1). Amplicon libraries for each of the four communities were prepared using a two‐step amplification protocol to minimize bias introduced by barcoded primers (Appendix S1: Supplemental Materials and Methods). The AOB, *Nitrospira* and *Nitrobacter* NOB communities were then pooled and sequenced in a single run on the MiSeq platform (v3 reagent kit, 2 × 300 bp paired‐end reads; Illumina, San Diego CA, USA), while AOA communities were sequenced on a Roche 454 FLX Genome Sequencer (Roche) using FLX+ chemistry due to the larger amplicon size. After postprocessing of raw reads, final sequence data sets for each gene were clustered at 97% nucleotide similarity using the UPARSE algorithm (Edgar, 2013). The use of the same bioinformatics pipeline for processing of OTUs at 97% should minimize potential differences between sequence data sets, in particular base‐calling error rates, obtained from Illumina and 454 platforms (Knight et al., 2018; Sinclair, Osman, Bertilsson, & Eiler, 2015) that may be problematic in network reconstruction. Representative sequences for OTUs of each gene were translated to amino acids and aligned to respective reference alignments using HMMER (Eddy, 1998), followed by back‐translation to nucleotide alignments. Phylogenetic mapping of representative OTU sequences using the evolutionary placement algorithm in raxml v8.0 (Stamatakis, 2014) was performed to examine the distribution of OTUs across the different reference phylogenies (Supporting information Figures S3‐S6). Representative OTU sequences that were similar to known outgroups in the phylogenies of each gene were considered nonspecific products, and the OTU was removed from the data set. The resulting number of pre‐ and postprocessing sequences and OTUs for each data set is shown in Supporting information Table S2. Based on rarefaction analysis, coverage at each sampling site was high for each of the four communities. (Supporting information Figure S2). Phylogenies of the final set of representative OTU sequences for each gene were calculated from nucleotide alignments using fasttree 2 (Price, Dehal, & Arkin, 2010), and classification of OTUs based on previously defined lineages within reference phylogenies for each gene (Supporting information Figures S3‐S6) was performed using the classifier in mothur (Schloss et al., 2009). A complete description of all PCR and thermal cycling conditions, as well as postprocessing of raw sequence reads and OTU clustering, is provided in Appendix S1: Supplemental Materials and Methods.

### 2.5 Diversity of AO and NOB communities

The richness (*S*) and evenness (1 – Simpson's *D*) of OTUs for AOA, AOB, *Nitrobacter* and *Nitrospira* functional groups were calculated using the “vegan” package in r, while phylogenetic diversity (PD; Faith, 1992) based on the resulting OTU phylogenies was calculated using phylocom (Webb, Ackerly, & Kembel, 2008). All diversity metrics were determined by taking the mean value of each statistic across 100 randomly rarefied OTU tables, with sampling size corresponding to the minimum number of sequences across the set of 51 samples for each group. Spearman correlations were calculated in R to examine relationships between the abundance and diversity of NOB communities with that of AOA and AOB.

### 2.6 Co‐occurrence of nitrite‐oxidizing bacteria and ammonia‐oxidizing bacteria and archaea

Analysis of general patterns of AO and NOB OTU co‐occurrence was performed on rarefied OTU tables using checkerboard scores (*C*‐score) tested against a null model that preserves both site (columns) and species (row) sums (Strona, Nappo, Boccacci, Fattorini, & San‐Miguel‐Ayanz, 2014). The standardized effect score (SES) was calculated as (*I*_o_ – *I*_s_)/*S*_s_ (Gotelli & McCabe, 2002), where *I*_o_ is the observed value of the index, and *I*_s_ and *S*_s_ are the mean and standard deviation, respectively, of the index for 9,999 simulated “null” communities. This value indicates if pairs of OTUs are more spatially segregated (values >2) or tend to aggregate more (values <−2) than expected by chance. *C*‐score and SES values were calculated for each OTU table individually, as well as for the combined data set of all four groups.

Network analysis was performed using the “igraph” package in r on the complete set of OTUs from all four AO and NOB functional groups to detect specific modules of co‐occurring ammonia and nitrite‐oxidizing lineages. Prior to analysis, OTUs occurring in <10% of sites were excluded. To account for differences in sampling depth, OTU count tables for each gene were transformed using the regularized log transformation in the “deseq2” package (Anders & Huber, 2010; Love, Huber, & Anders, 2014). Co‐occurrences of all OTUs across the samples sites were then determined by calculating Pearson correlations on the matrix of concatenated, transformed OTU tables. As microbial association networks typically assume a scale‐free topology, that is, there are few highly connected groups (“hub” nodes) with a large number of groups having few connections (Barberán et al., 2012; Faust & Raes, 2012; Ma et al., 2016), we selected a correlation threshold of *r *≥* *0.64 based on the fit of the degree distribution to a power law (*R* ^2^ = 0.9) using the “pickHardThreshold” command in the “wgcna” package in r (Langfelder & Horvath, 2008). Negative correlations were excluded, as we were interested in detecting communities of co‐associated ammonia and nitrite‐oxidizing OTUs across the field site. All remaining correlations were found to be significant after correcting for false discovery rate (*p* < 0.001), and unconnected nodes (degree = 0) were excluded. After removal of OTUs that were infrequently detected across the sites, the final network consisted of 520 nodes and 2,293 edges, with a global network diameter of 18 and an average node degree of 8.8, and was visualized in Cytoscape using the Fruchterman–Reingold algorithm (Fruchterman & Reingold, 1991).

### 2.7 Identification and analysis of nitrifier community modules

The “infomap” algorithm (Rosvall & Bergstrom, 2008) was used to detect distinct modules of ammonia and nitrite‐oxidizing lineages in the final graph. Community modules with more than 5 nodes were used in eigengene analysis, where the matrix of transformed OTU abundances by sites for the specific set of OTUs in a given module was decomposed to a single eigenvector using singular value decomposition (Langfelder & Horvath, 2008). A main advantage of this analysis is that rather than focusing on shifts in the abundance of individual OTUs, higher‐order organization can be examined in the networks structure, and potentially important edaphic factors that differentiate co‐occurring AO/NOB lineages into subcommunities (modules) can be identified (Deng et al., 2012). As we only used positive correlations, eigenvectors reflect similar shifts in the overall abundance of OTUs in each module detected by “infomap” and were compared to environmental variables as well as each other by Spearman correlations and cluster analysis. For all modules, the variance explained by each eigenvector ranged between 0.85 and 0.98, indicating that information loss was minimal. The complexity of the different modules was compared by calculating the average intramodular degree (*k*_in_), which is the mean number of connections between adjacent nodes within the same module (Carlson et al., 2006; ). The specificity of different AOA, AOB, *Nitrobacter* NOB and *Nitrospira* NOB lineages to each module was determined by calculating network assortativity, which quantifies the tendency for nodes with similar properties—in this case lineage classification of OTUs—to connect to each other. The significance of both network modularity and assortativity was determined using the “rewire.edges” command in the “igraph” package (Csardi & Nepusz, 2006), in which the modularity score of the observed network was compared to the modularity scores of 999 randomly rewired networks, each preserving the original degree distribution.

Recent work has shown that different methods of network inference vary in their performance and limitations (Weiss et al., 2016). To assess the robustness of our initial network, we performed three additional network inferences using different methods (Appendix S1: Supplemental Materials and Methods). First, we used random matrix theory (RMT; Deng et al., 2012) to identify a Pearson correlation threshold for the regularized log‐transformed OTU abundance data, as this method does not assume scale‐free network topology. Second, the SparCC algorithm (Friedman & Alm, 2012) was used on OTU tables that were transformed to absolute abundance data by multiplying the proportion of each OTU within a functional group by the total abundance of that group, as determined by qPCR. Finally, each of the four OTU tables was rarefied, followed by calculation of absolute OTU abundances as done for the SparCC analysis. Spearman correlations were then calculated and RMT was used to determine the correlation threshold. The co‐occurrences of different functional group lineages within each network were compared visually, and the modularity, assortativity and Jaccard similarity of each network were compared to the original network (Appendix S1: Supplementary Results, Table S3, Figures S7‐S11). Based on these comparisons, we used the initial network structure and modules for subsequent analyses.

### 2.8 Geostatistical analysis and mapping

The presence of spatial autocorrelation in the abundance and diversity of *Nitrospira* and *Nitrobacter* NOB communities, as well as the eigenvector scores of modules of co‐occurring AO/NOB OTUs across the entire farm, was assessed using Moran's *I* index (Moran, 1950). Gene abundance data were log‐transformed prior to analysis, while module eigenvectors were rank transformed. Variables that exhibited significant spatial structure (*Z*‐score >1.96; *p *<* *0.05) were then modelled geostatistically using the “gstat” package in r (Pebesma, 2004). Resulting variograms were evaluated using leave‐one‐out cross‐validation, and the root‐mean‐squared error of prediction (RMSEP), correlation between observed and predicted values (*r*), and the ratio of performance deviation (the standard deviation of the variable divided by the RMSEP) were used to evaluate prediction error. Final variogram parameters were adjusted manually if prediction was improved based on cross‐validation results. Predicted values of gene abundances were then back‐transformed from log‐based values, whereas predicted module eigenvector scores were back‐transformed from rank‐adjusted values according to Wu, Norvell, and Welch (2006). Interpolation and mapping were performed using ordinary kriging and plotting functions within the “gstat” and “lattice” packages in r.

## 3 RESULTS

### 3.1 Phylogenetic association of *nxrB* and *amoA* genes

Communities of AOA consisted of lineages within the *Nitrososphaera* cluster, with only one OTU of low abundance (<0.01%) grouping with *Nitrosotalea* (Supporting information Figure S3). This lineage is characterized as being acidophilic; however, known isolates grow at pH up to 5.5 (Lehtovirta‐Morley, Stoecker, Vilcinskas, Prosser, & Nicol, 2011), and the lowest pH value observed in the field was 5.7. Over 60% of reads mapped to lineages within *Nitrososphaera* subclusters 8 and 11 and *Nitrososphaera* sister subclusters 1 and 2 (Pester et al., 2012). For the AOB, 72% of the *amoA* sequences were most similar to *Nitrosospira* cluster 3a *amoA* genes (Purkhold et al., 2003), with one dominant OTU (63% of all AOB *amoA* sequences) similar to a strain found in an enrichment culture generated from fertilized pasture land in the United Kingdom (Supporting information Figure S4), which was observed to be sensitive to high ammonia concentrations (>70 mM NH_4_^+^; Tourna, Freitag, & Prosser, 2010). Approximately 27% of AOB sequences belonged to *Nitrosospira* cluster 2a, and <1% were related to *amoA* from *Nitrosomonas*, mainly *Nitrosomonas oligotropha/urea*.

The *Nitrospira nxrB* OTUs were highly diverse, covering a range of different clades within the reference phylogeny (Supporting information Figure S5). Over 27% of reads grouped within lineage II near the “Austrian forest soil cluster” (Pester et al., 2013), whereas another 24% and 15% were most similar to the “Namibian soil cluster 2” and *Nitrospira* lineage I, respectively. Less than 1% of reads were classified as the lineage II group containing the comammox species *Nitrospira inopinata*; however, phylogenetic mapping showed 12% of reads being more similar to this subclade than others within lineage II. In addition to the described clades of *Nitrospira nxrB*, we observed a distinct clade of *nxrB* sequences obtained from soil and groundwater metagenomes to which 15% of *Nitrospira nxrB* reads were associated. For the *Nitrobacter*, >80% of the reads were most similar to *nxrB* from a clade that is closely related to *Nitrobacter vulgaris*, with over 67% of reads grouping in a single OTU (Supporting information Figure S6). However, 7% of reads were associated with more divergent lineages found in soil metagenomes, while 2% were associated with *Nitrobacter hamburgensis*. Only one low‐abundant OTU (<0.01%) was found to be most similar to *nxrB* from *Nitrolancetus hollandicus*, within the Chloroflexi.

### 3.2 Spatial distribution of abundance and diversity of AO and NOB

**Figure 1:**
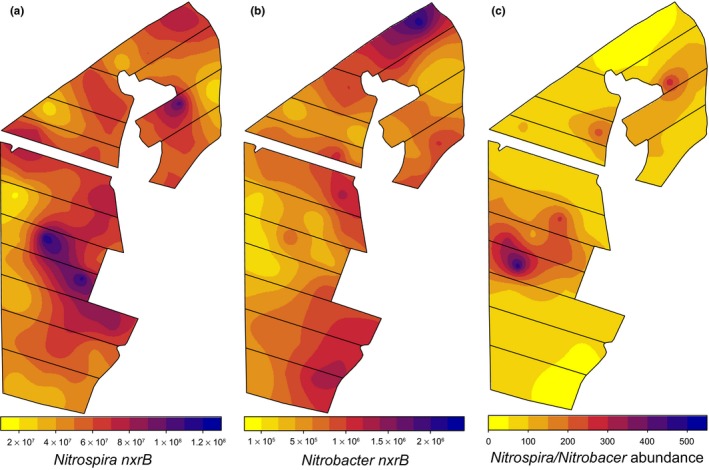
Kriged maps showing the spatial distribution of *Nitrobacter nxrB* and *Nitrospira nxrB* gene abundance across the field site. (a) *Nitrospira nxrB* and (b) *Nitrobacter nxrB* abundance (copies per g soil dw). (c) Ratio of *Nitrospira* to *Nitrobacter nxrB* gene abundance

*Nitrospira* was the dominant nitrite‐oxidizing community, with *nxrB* copies ranging from 1.8 to 12 × 10^7^ copies per g soil dry weight. By contrast, the abundance of *Nitrobacter nxrB* was 30–500 times lower than that of *Nitrospira nxrB*, varying from 0.9 to 23 × 10^5^ g^−1^ soil dry weight. The total abundance of nitrite‐oxidizing communities, as measured by qPCR of *nxrB* genes, was comparable to that of ammonia‐oxidizing communities reported in Wessén et al. (2011), with the ratio of total *amoA* (AOA + AOB) to total *nxrB* (*Nitrospira* + *Nitrobacter*) gene copies varying from 0.26 to 3.55. However, clear differences in the association of AO and NOB communities were observed, as the abundance of *Nitrospira nxrB* was positively correlated to that of AOA *amoA*, but not AOB (Table 1). Similarly, the abundance of *Nitrobacter nxrB* was positively correlated with AOB *amoA* gene copy number, with no significant relationship to that of AOA. Significant spatial dependence was observed for the total abundance of both NOB communities as well as the *Nitrospira*:* Nitrobacter* ratio (Supporting information Table S4). Although both communities were lower in abundance in the western part of the integrated field area, they largely exhibited differential spatial patterns across the farm (Figure 1a,b). This is reflected in the ratio *Nitrospira* to *Nitrobacter nxrB* genes, with the highest values in the central areas of the integrated farming system (Figure 1c). This region also had the highest ratio of AOA to AOB reported by Wessén et al. (2011), in agreement with the contrasting correlations observed for AOA, AOB and NOB communities (Table 1).

**Table 1:** Spatial autocorrelation (Moran's *I* test) and Spearman's correlations (ρ) of the diversity (*S* and *D*, OTU richness and Simpson's evenness, respectively; PD, Faith's phylogenetic diversity) and abundance (q*nxrB*) of *Nitrobacter* and *Nitrospira* nitrite‐oxidizing bacterial communities with the diversity and abundance (q*amoA*) of ammonia‐oxidizing archaea (AOA) and bacteria communities (AOB)

	*Nitrobacter* NOB				*Nitrospira* NOB			
	*S*	*D*	PD	q*nxrB*	*S*	*D*	PD	q*nxrB*
Spatial pattern								
Moran's *I* a	2.88*	2.72*		1.94*				2.19*
AOA								
*S*	0.32**		0.28*		0.44**		0.44**	0.31*
*D*	0.28*	0.41**		−0.36*	0.31*			
PD	0.41**		0.35*		0.34*		0.31*	0.38**
q*amoA*	0.28*				0.34*		0.35*	0.42**
AOB								
*S*	0.37**	0.41**	0.38**			0.28*		−0.36**
*D*	0.59***	0.45**	0.53***					
PD	0.37**		0.37**					
q*amoA*				0.38**	−0.28*		−0.31*	

Notes

BD: bulk density; DW: dry weight; DON: dissolved organic nitrogen; DOC: dissolved organic carbon.

*0.01 < *p *<* *0.05; **0.001 < *p *<* *0.01; ***p <* *0.001.

a *p*‐values based on two‐sided permutation test, 9,999 permutations.

With respect to diversity, only *Nitrobacter* OTU richness and evenness exhibited significant spatial autocorrelation across the farm (Table 1). The diversity of both NOB communities was positively correlated to that of AOA, with the strongest association observed between AOA and *Nitrospira* richness. However, nearly all measures of *Nitrobacter* diversity were positively, and in some cases quite strongly, correlated to AOB diversity, whereas only *Nitrospira* evenness was associated with the richness of AOB communities. Furthermore, *Nitrospira* OTU richness and PD were positively correlated to the abundance of AOA communities, yet were negatively associated with the abundance of AOB.

### 3.3 Co‐occurrence analysis of ammonia and nitrite‐oxidizing communities

All communities exhibited significant nonrandom patterns of co‐occurrence based on checkerboard scores (Table 2), with positive standardized effect scores (SES) indicating that OTUs were more segregated (i.e., co‐occurred less frequently) across the sampling sites than expected by chance for each individual functional group, as well as for the combined communities. However, the degree of segregation varied between the different groups, as *Nitrospira* and AOA OTUs had higher *C*‐scores and were substantially more segregated based on SES values than *Nitrobacter* and AOB OTUs, respectively. The combination of OTUs from all groups resulted in an even higher degree of segregation and both *C*‐score and SES values increased when considering only the OTUs that were identified as strongly associated based on our co‐occurrence threshold (*r *≥* *0.64).

**Table 2:** Checkerboard scores (*C*‐score) and standardized effect size (SES) quantifying the co‐occurrence of ammonia and nitrite‐oxidizing community OTUs across the Logården field site

Community	*C*‐score^a^	SES
AOA	37.0	10.2
AOB	18.5	4.5
*Nitrobacter* NOB	21.5	4.4
*Nitrospira* NOB	33.0	23.6
Total nitrifier community	31.5	31.8
Total nitrifier community, *r* ≥ 0.64	40.0	58.3

a *p* < 0.001 for all values based on null model with preserved row and column sums.

### 3.4 Network analysis of ammonia and nitrite‐oxidizing communities

**Figure 2:**
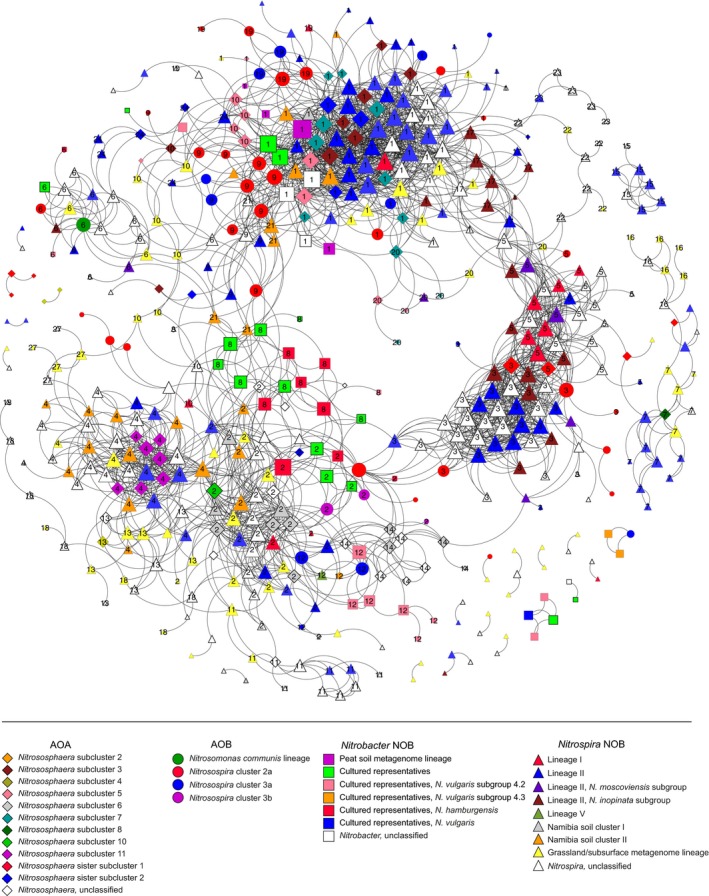
Network analysis of co‐occurring AOA, AOB, *Nitrospira* and *Nitrobacter* OTUs based on Pearson correlations (*r*). Complete network of all AO and NOB OTUs with degree >0. Node size is proportional to node degree, while connections between each node indicate significant positive correlations above the set threshold (*p* < 0.01, *r *≥* *0.64). The shape and colour of the nodes denote the functional group and lineage, respectively, of each OTU, while the numbers indicate membership of co‐occurring OTUs to distinct community modules

We further examined the co‐occurrence of AO and NOB OTUs by network analysis (Figure 2). In the network topology, 25 modules ranging from 5 to 93 co‐occurring OTUs were identified, resulting in a significant modularity score of 0.69 (*p *<* *0.001). Comparison of this inference with networks generated using the methods described above showed consistent patterns of lineages from different functional groups forming distinct modules within each network structure (Supporting information Figures S7, S9 and S10; Appendix S1: Supplemental Results), with comparable modularity scores (Supporting information Table S3). Within the network, five main clusters of AO and NOB OTUs are apparent (Figure 2). Module 1 was the most diverse with OTUs from all four functional groups. The complexity of module 1, as measured by average number of intramodule connections between nodes (*k*_in_), was higher than that of the complete network, with *k*_in_ = 14.6. By contrast, module 2 also included all four groups, yet was less complex (*k*_in_ = 8.9). Modules 3 and 5 both lacked *Nitrobacter* NOB OTUs but differed in their complexity (*k*_in_ = 16.4 and 9.0, respectively), whereas module 4 consisted of only AOA and *Nitrospira* OTUs and was only slightly higher in complexity than the overall network (*k*_in_ = 10.7). The different lineages of AOA, AOB and NOB within each module showed varying levels of module‐specific association, with a significant network assortativity score of 0.19 (*p *<* *0.001) indicating that OTUs within the same lineage tended to be more connected than by chance. Modules with similar compositions of AO and NOB OTUs were also detected in networks inferred using alternative methods (Supporting information Figures S7–S11), also with significant levels of assortativity (Supporting information Table S3). In agreement with the *C*‐scores, *Nitrospira* and AOA lineages showed the largest degree of segregation between different modules. Different subgroups of *Nitrospira* lineage II were found in all of the larger modules within the network, whereas *Nitrospira* lineage I was more prevalent in module 5, one of only two modules with the *Nitrososphaera* sister subcluster 1 lineage of AOA. OTUs similar to that of *Nitrospira inopinata*, capable of complete ammonia oxidation to nitrate, were also more prevalent in modules 5 and 3, as well as in a smaller module, 17, in close proximity in the network. Within the AOA, *Nitrososphaera* subclusters 3 and 7 were only found in modules 1 and 20, which were closely associated in the network, while *Nitrososphaera* subclusters 8, 10 and 11 were only found within modules 7, 2 and 4, respectively (Figure 2). Lineages of AOB were less module‐specific, although the most abundant AOB OTUs, *Nitrosospira* cluster 3a, were predominantly found in modules 9 and 19 in close association with module 1. The only *Nitrosomonas* OTUs in the network belonged to the *N. communis* lineage, and this AOB was a central node in module 6. nitrite‐oxidizing communities were also dispersed across the different modules; however, module 8 consisted exclusively of *Nitrobacter* lineages similar to that of cultured representatives, whereas more divergent *Nitrobacter* OTUs similar to sequences obtained from peat soil metagenomes were only found in module 1.

### 3.5 Spatial distribution of nitrifier community modules in relation to edaphic factors

**Figure 3:**
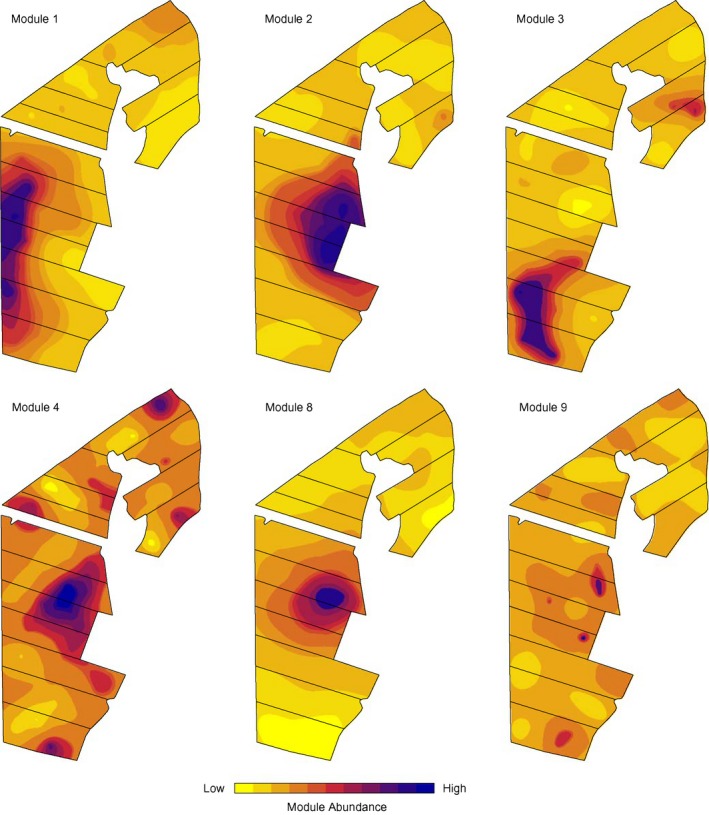
Kriged maps showing the distribution of nitrifier community modules exhibiting significant spatial autocorrelation across the field site (Supporting information Table S3). Scale reflects overall abundance of OTUs in each module based on eigenvector scores at each sampling location

**Figure 4:**
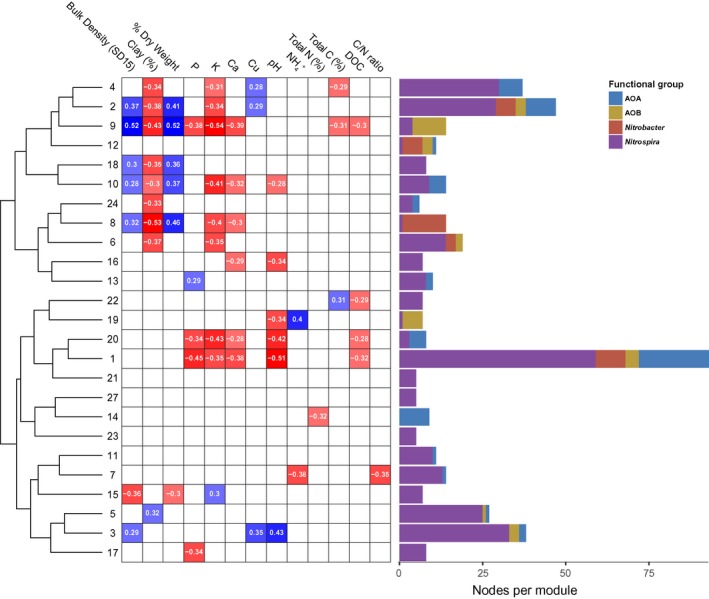
Heatmap showing significant correlations (Spearman's ρ, *p* < 0.05) between soil properties and the eigenvalues of different nitrifier community modules. Blue cells show significant positive relationships between overall module abundance and soil properties, while red cells denote negative relationships. Clustering of modules is based on Spearman correlations between the eigenvalues of each community module. Horizontal bars to the right show the number of nodes from AOA, AOB, *Nitrospira* NOB and *Nitrobacter* NOB functional groups in each module

Significant spatial dependence was observed for the larger modules 1–4 and 8 and 9 (Figure 3). Cluster analysis showed a clear pattern of modules with similar spatial distributions being significantly correlated to similar sets of edaphic factors (Figure 4). Comparison of module abundance with edaphic factors (Figure 4) showed that the decrease in module 1 abundance corresponded to increased pH and P, K, Ca and dissolved organic carbon content. In contrast, the abundance of modules 2, 4, 8 and 9 was higher in sampling locations with lower clay and K content. Interestingly, the abundances of modules 8 and 9, which consisted largely of *Nitrobacter* NOB and *Nitrosospira* cluster 2a OTUs, respectively (Figure 2), were also highest in the low‐clay central region of integrated farm yet, unlike module 4 in the same region, was significantly correlated to soil bulk density and moisture content (Figure 4). Module 3 abundance was highest in the southern region of the integrated farmed area, corresponding to increased soil pH, Cu and soil bulk density. This region was also reported to have a higher abundance of AOB *amoA* (Wessén et al., 2011) than the central region of the integrated farm.

## 4 DISCUSSION

Our results show that when nitrification is a shared pathway between two functional groups, the partnership is not random. At the functional group level, *Nitrospira* and *Nitrobacter* NOB communities had contrasting distributional patterns across the 44‐ha area, similar to the spatial distributions of AOA and AOB communities reported in Wessén et al. (2011) (Figure 1). Thus, the abundance of *Nitrospira* NOB was positively correlated to that of AOA, whereas *Nitrobacter* NOB abundance was correlated only to that of AOB. Similar results have been observed in grasslands (Simonin et al., 2015; Stempfhuber et al., 2016) and forest soils (Stempfhuber et al., 2017); however, Ke et al. (2013) reported high abundances of AOA and *Nitrobacter* in bulk soils of rice paddies. It is hypothesized that *Nitrobacter* are *r*‐strategists, preferring higher NO_2_^−^ and oxygen concentrations, whereas *Nitrospira* species are K‐strategists that thrive in low NO_2_^−^ conditions and at oxic–anoxic interfaces (Attard et al., 2010; Daims, Nielsen, Nielsen, Schleifer, & Wagner, 2001; Schramm, de Beer, Gieseke, & Amann, 2000; Simonin et al., 2015; Wertz, Leigh, & Grayston, 2012). That *Nitrobacter* NOBs are more associated with AOBs and *Nitrospira* NOBs with AOAs fits with the theory that archaeal ammonia oxidizers have an advantage during low ammonia supply, although they are not restricted to these conditions (Hink, Gubry‐Rangin, Nicol, & Prosser, 2018; Sterngren, Hallin, & Bengtson, 2015). Other edaphic factors, such as oxygen and organic C availability (Le Roux et al., 2016), may also contribute to differential patterns of NOB distribution.

Nonrandom co‐occurrence patterns were also detected between lineages of the four functional groups. We know of no study that has explored whether such patterns exist within taxonomically constrained functional groups, and whether such patterns differ between functionally equivalent groups, but studies of microbial assemblages with broad taxonomic diversity show that nonrandom co‐occurrence patterns are commonly observed, often with more segregated (less co‐occurrence) structure than expected by chance (Barberán et al., 2012; Horner‐Devine et al., 2007; Jeanbille et al., 2016; Koenig et al., 2011). We observed that nitrifier communities were also more segregated than expected by chance and that AOA and *Nitrospira* were even more segregated across the farm than AOB and *Nitrobacter*. Significant patterns of segregation are commonly attributed to deterministic forces, such as competition, nonoverlapping niches or historical effects such dispersal limitation or evolutionary processes (D'Amen, Mod, Gotelli, & Guisan, 2017; Horner‐Devine et al., 2007). Both *Nitrospira* and AOA are known to be highly diverse functional groups that occur in a wide range of environments (Daims et al., 2016; Hatzenpichler, 2012; Pester et al., 2012, 2013), and niche partitioning across or even within lineages has been demonstrated for both groups (Gruber‐Dorninger et al., 2015; Gubry‐Rangin et al., 2011). For example, previous work in biofilms has shown that *Nitrospira* lineage I is more competitive in environments with higher NO_2_^−^ concentrations than lineage II (Maixner et al., 2006). As AOA and *Nitrospira* were the dominant and most diverse ammonia and nitrite‐oxidizing groups at the farm, the higher SES observed for these group likely reflects a larger range of niches compared to *Nitrobacter* and AOB.

While niche partitioning undoubtedly plays an important role, the assortative grouping of different lineages across the local communities (i.e., modules) may arise from shared ecophysiological characteristics or mutualistic interactions that are specific to each module. The complexity of each of the modules in the nitrifier co‐occurrence network varied substantially, with modules 1 and 3 being the most complex. More connections indicate a low degree of specialization among members within each local community and a shared niche between functional groups in these modules. For example, module 19 was dominated by the AOB lineages and, as expected, correlated with soil NH_4_^+^ content. Moreover, *Nitrospira* lineage I OTUs, which are competitive *Nitrospira* NOBs in environments with higher NO_2_^−^ concentrations (Maixner et al., 2006), were most prevalent in module 5 and co‐occurred with AOA OTUs within the *Nitrososphaera* “sister cluster” (Figure 2). Isolates and enrichment cultures of this previously undefined AOA lineage have recently been shown to prefer neutral pH conditions and can tolerate NH_3_ and NO_2_^−^ concentration levels typically associated with AOB (Lehtovirta‐Morley et al., 2016; Sauder et al., 2017). Other notable patterns, such as the co‐occurrence of *Nitrososphaera* subcluster 11 and the Namibian soil lineage of *Nitrospira* exclusively in module 4, may also be of potential significance from an ecophysiological perspective, especially considering the significant positive correlation of the abundance of this module with soil Cu concentrations. Unless an indirect relationship due to shared habitat preference, this could be a case of shared siderophore production and uptake mechanisms between AO and NOB groups described in Daims et al. (2016). However, further studies of pure or enrichment cultures are required to test this hypothesis.

Local communities were characterized by multiple connector nodes between modules but few hubs (Supporting information Table S3), although the identification of peripheral, module connector and module‐hub nodes fluctuated depending on the network analysis. This may indicate the presence of false‐positive edges or indirect interactions, and highlights the sensitivity of node classification to the method used for network inference. However, the higher proportion of module connector to module‐hub nodes observed across the different inference methods suggests a general ecological pattern. Module hubs and connectors are typically defined as being ecological specialists and generalists, respectively (Olesen, Bascompte, Dupont, & Jordano, 2007). Although this interpretation is highly dependent on the degree to which niche overlap explains network structure (Faust & Raes, 2012), a modular network with a greater proportion of generalists to specialists may indicate communities that have been largely shaped by disturbances (Hawkes & Keitt, 2015). Experimental and theoretical studies have indicated that community modularity can buffer the effect of disturbances by allowing for a larger metacommunity (Gilarranz, Rayfield, Liñán‐Cembrano, Bascompte, & Gonzalez, 2017; Stouffer & Bascompte, 2011). Agroecosystems are defined by recurring disturbance events; thus, the preponderance of “generalist” connector over “specialist” hub nodes may reflect nitrifier community assemblages that are adapted to fluctuating conditions.

The abundance of the most complex modules increased in the area under integrated management (Figure 3). This area receives inputs of fertilizer N and overall also higher N input compared to the northern, organically managed area where N_2_‐fixation was the main source of added N (Stenberg et al., 2012). The availability of resources can be an important driver of network complexity at scales ranging from the rhizosphere (Shi et al., 2016) to landscapes (Ma et al., 2016), and this could potentially explain the observed differences. However, high N loadings can also be viewed as a disturbance and theory predicts that more complex communities, that is, those with high connectivity between taxa within and between modules, will exhibit greater stability in ecosystems that are more frequently disturbed (Mougi & Kondoh, 2012). The highest abundance of module 3, being the most complex module of them all, was found in the southern‐most area (Figure 3c). Previous reports on N‐cycling communities at our study site have shown that this area had the lowest ratio of AOA to AOB across the field (Wessén et al., 2011), as well as the highest abundance of genes associated with anaerobic denitrifiers and non‐denitrifier N_2_O‐reducing communities (Enwall et al., 2010; Juhanson, Hallin, Söderström, Stenberg, & Jones, 2017;). Moreover, potential ammonia oxidationand denitrification activities were highest in this region indicating that this area is a “hotspot” of N‐cycling. Nevertheless, it had the lowest NO_3_^−^ leaching rates (Stenberg et al., 2012) which together with the other findings suggest N loss through gaseous emission rather than leaching, driven by the specific nitrifying communities dominating this area. This indicates that tightly coupled processes prevent accumulation of nondesired intermediates such as NO_3_^−^. Future work should focus on the overall N‐transforming network rather than individual functional groups, as local reaction rates may largely depend on interacting microorganisms across or within pathways.

In conclusion, our results highlight the usefulness of network analysis for providing insight into the factors that drive the spatial distribution of functional groups known to interact within soil nitrifying communities. While we observed significant associations of broadly defined functional groups, closer analysis of co‐occurrence patterns shows that substantial physiological variation may exist among lineages that defies broad‐brush descriptions of an entire functional group. By identifying specific modules of co‐associated AO and NOB lineages, we were able to identify putative niches and potential interactions in a spatial context that more accurately reflects this variation. The ecophysiology described for a few nitrifiers in pure culture was supported in our analysis, demonstrating that network inference combined with external data can place information obtained from pure cultures into an ecological context. Although network analysis does not explain causal mechanisms (Röttjers & Faust, 2018), these findings provide useful starting points for future manipulation experiments that can confirm, or refute, inferences on lineage‐specific interactions and mechanisms underlying nonrandom associations within and between functional groups and entire N‐transforming networks.

## AUTHOR CONTRIBUTIONS

C.M.J. and S.H. designed the study. C.M.J. performed all laboratory work and data analysis. The manuscript was written by C.M.J. and S.H.

## Data Availability

The complete sequence data set is available in the NCBI Short Read Archive under BioProject Accession no. PRJNA436119. Quantitative PCR, diversity metrics and soil data as well as OTU tables are provided in Supporting information Appendix.
