# Supplementary material for: Geospatial variation in co‐occurrence networks of nitrifying microbial guilds
Source: Mol Ecol. 2018 Nov 3;28(2):293–306. doi: 10.1111/mec.14893 (PMC6905385; doi:10.1111/mec.14893)
Supplement: Supplementary file 1 [file MEC-28-293-s001.zip › mec14893-sup-0001-Supinfo.pdf]

Supplemental Information for:

Geospatial variation in co-occurrence networks of nitrifying microbial guilds

Christopher M. Jones and Sara Hallin

Table of Contents:

**Supplementary methods**	Page 1
**Supplementary results**	Page 4
**Table S1**	Page 6
**Table S2**	Page 7
**Table S3**	Page 7
**Table S4**	Page 8
**Figure S1**	Page 9
**Figure S2**	Page 10
**Figure S3**	Page 11
**Figure S4**	Page 12
**Figure S5**	Page 13
**Figure S6**	Page 14
**Figure S7**	Page 15
**Figure S8**	Page 16
**Figure S9**	Page 17
**Figure S10**	Page 18
**Figure S11**	Page 19
**References**	Page 20

Supplementary Methods

Generation of reference nxrB *and* amoA *gene alignments and phylogenies*

Reference sequences for each gene were acquired from previously compiled datasets (AOA, Pester *et al*., 2012; AOB, Purkhold *et al*. 2000, Purkhold *et al*. 2003, Mintie *et al.* 2003; *Nitrospira nxrB*, Pester *et al*. 2013, Daims *et al.* 2015, van Kessel *et al.* 2015; *Nitrobacter nxrB*, Vanparyes *et al*. 2007). Additional sequences for AOB *amoA* and *nxrB* genes were obtained by generating positional site-scoring matrices from amino acid alignments for use in PSI-TBLASTN searches of draft and complete genomes obtained from NCBI, as well as assembled metagenome datasets downloaded from the Integrated Microbial Genomes database (Markowitz *et al.* 2012). Redundancy in the datasets was reduced by clustering at 99 % nucleotide similarity, followed by selection of a single representative sequence for each cluster. Both AOB and *Nitrobacter nxrB* reference sequences were aligned by amino acid using the MAFFT-LINSI algorithm (Katoh & Standley 2013), followed by manual correction. The final alignment of *Nitrospira nxrB* reference sequences was generated using HMMER (Eddy 1998) based on the alignment in Pester *et al*. (2013), followed by manual alignment and mapping of nucleotides to amino acid positions in ARB (Ludwig *et al.* 2004).

The phylogeny from Pester *et al*. (2012) was used for phylogenetic mapping and classification of AOA *amoA* reads. Reference phylogenies for AOB *amoA* and *Nitrobacter nxrB* genes were generated from nucleotide alignments using ExaBayes v1.4 (Aberer *et al.* 2014) with the GTR+Γ substitution model (Waddell & Steel 1997). Inferences were performed using two separate runs of four MCMC chains each, and run for 13 and 3.2 million generations for AOB *amoA* and *Nitrobacter nxrB,* respectively, resulting in standard deviation of split frequencies below 0.01 for all trees. Due to failure of Exabayes to converge on the final tree topology, the *Nitrospira nxrB* reference phylogeny was based on maximum likehood search using IQTREE, with node support determined using ultrafast bootstrapping (Nguyen *et al.* 2015; Hoang *et al.* 2018). Lineages were defined in each phylogeny (Figs. S3-S6) based on classifications in previous studies (AOA *amoA*, Pester *et al.* 2012; *Nitrospira nxrB*, Pester *et al.* 2013; AOB *amoA*, Purkhold *et al.* 2000; Avrahami *et al.* 2002; Purkhold *et al.* 2003); *Nitrobacter nxrB*, Vanparys *et al.* 2007), as well as by identifying distinct, well-supported clades (Bayesian posterior probabilities > 0.9; Bootstrap probabilities > 80%) within the phylogenies generated in this study that have not been described previously.

Amplification and sequencing of AOB amoA*, Nitrospira* nxrB, and *Nitrobacter* nxrB *genes using the MiSeq platform*

AOB *amoA*, *Nitrospira nxrB*, and *Nitrobacter nxrB* genes were first amplified in duplicate 25 µL reactions for each sample using 1X Phusion HF master mix, 0.2 µg/µL BSA (New England Biolabs), and 0.5 µM group specific primers with Nextera XT (Illumina, San Diego CA, USA) adaptors for forward (5'-TCG TCG GCA GCG TCA GAT GTG TAT AAG AGA CAG NNN N-3') and reverse (5'-GTC TCG TGG GCT CGG AGA TGT GTA TAA GAG ACA G-3') primers. A pad of four random 'N' bases was placed between the forward adaptor and the group specific primer to improve cluster detection during the initial sequencing cycles. Primer sets and thermal cycling conditions used to amplify each group are described in Table S1. For AOB *amoA* and *Nitrospira nxrB* genes, 1.5 mM $MgCl_2$ and 15 ng soil DNA extract were used in each reaction, whereas 2.5 mM $MgCl_2$ and 25 ng soil DNA extract were used to amplify *Nitrobacter nxrB.* The resulting duplicate products were pooled for each sample and examined by agarose gel electrophoresis, then purified using AxyPrep Mag™ PCR clean-up kit (Axygen Biosciences, Union City CA, USA). Nextera XT Sequencing adaptors and dual-index barcodes were then added to each amplicon library via a second amplification step in which 3 µL of purified product was used as template in a 50 µL reaction consisting of 1X Phusion master mix and 0.2 µM barcoded forward and reverse sequencing primers. Thermal cycling conditions for the second PCR step for all three libraries consisted of an initial denaturing step at 98 °C for 3 min, followed by 8 cycles of 98 °C 30 sec, 55 °C 30 sec, and 72 °C for 45 sec, with a final extension step of 72 °C for 5 min. For the *Nitrobacter nxrB*, the number of cycles was increased to 10 cycles to increase yield. The final barcoded products were inspected by electrophoresis and quantified using the Qubit dsDNA high sensitivity assay (HS) kit. All products were then sent to Microsynth AG (Balgach, Switzerland) for sequencing in a single MiSeq run using 2 x 300 bp chemistry, providing an overlap of 100, 180, and 220 bp for paired *Nitrospira nxrB*, AOB, and *Nitrobacter nxrB* reads, respectively.

Due to the size of the fragment (629 bp), sequencing of AOA *amoA* genes was performed using the Roche 454 pyrosequencing platform with FLX+ Titanium chemistry. Samples were prepared using the two-step procedure outlined in (Berry *et al.* 2011) to minimize bias due to use of barcoded primers. An initial amplification step of 25 cycles was performed in duplicate 25 µL PCR reactions using AOA specific primers without barcodes for each sample with 0.8 µM of each primer, 0.2 µg/µL BSA (New England Biolabs), 1X Phusion master mix with 1.5

mM $MgCl_2$ (Thermo Fisher Scientific, Stockholm, Sweden), and 15 ng soil DNA extract. Primer sets and thermal cycling conditions are described in Table S1. The resulting products were then pooled and inspected by agarose gel electrophoresis, and 2 µL of product was used as template in a second amplification step of 6 cycles under the same thermal cycling conditions in 50 µL reaction volume, containing 0.2 µM barcoded primers with 454 Lib-L adaptors and 1X Phusion master mix. Final barcoded products were then inspected by agarose gel electrophoresis and quantified using the Qubit 1.0 in combination with the dsDNA HS assay, then sent to Microsynth for sequencing.

Pre-processing and OTU clustering of Illumina and 454 reads

Paired AOB-*amoA*, *Nitrospira nxrB*, and *Nitrobacter nxrB* reads were merged using PEAR (Zhang *et al.* 2014). Reads were trimmed at points where at least two consecutive bases had quality scores less than 30, and only reads with at least 50 bp overlap were merged. Sequences less than 300 bp or greater than 500 bp were excluded. The remaining sequences were then quality filtered using the 'fastq_filter' command in USEARCH v8 (Edgar 2010), and those with a maximum expected error score above 1 were removed. The AOA *amoA* reads were demultiplexed and quality filtered using QIIME (Caporaso *et al.* 2010), with reads below an average quality score of 25 removed. All datasets were then screened using HMM-FRAME (Zhang & Sun 2011) to remove non-specific amplicons and correct for frameshift errors, using HMMs generated from the corresponding reference alignments. The UPARSE algorithm (Edgar 2013) was used to remove potential chimeras, using the ARB databases as references for the reference-based chimera-checking step, followed by clustering into OTUs. We chose a cutoff of 97 % similarity to determine OTUs for all four datasets as this level reduces the chance of artificially inflating diversity due to the presence of sequencing errors (Kunin *et al.* 2010) while at the same time allowing for the retention of higher phylogenetic resolution that may be functionally relevant (Gruber-Dorninger *et al.* 2015). The minimum number of sequences per OTU was set at two, thus singletons were excluded in subsequent steps. For each group, OTUs were then classified using the naïve Bayesian classifier (Wang *et al.* 2007) implemented in the 'classify.seqs' function in MOTHUR (Schloss *et al.* 2009), using the reference datasets and lineage assignments described above to train the classifier.

Additional network analyses using random matrix theory, SparCC, and Spearman correlations of rarefied tables

To assess the robustness of the network analysis described in the article, we performed additional network inference using three different methods and compared the resulting networks with our original inference (Figure 2). First, random matrix theory (RMT, Deng *et al.*, 2012) implemented in the 'RMThreshold' package (Menzel, 2016) was used to identify potential threshold *r*-values in the initial Pearson adjacency matrix. This method does not assume a scale-free network topology, but rather determines a threshold value at which non-random properties of a matrix emerge, based on fitting the nearest neighbor spacing distribution of eigenvalues to Gaussian orthogonal ensemble or Poisson distributions. The implementation of RMT used here provides a range a threshold values depending on the criterion for evaluating the fits to the different distributions, and the value selected for the final network (Figure S7) was based the combination of these criteria (Figure S8). Second, we determined the absolute abundances of OTUs for each group by multiplying OTU proportions by the respective qPCR data obtained for each group, followed by analysis of the concatenated dataset using SparCC (Friedman & Alm, 2012). Correlations were determined using 100 iterations of 20 internal iterations, and pseudo P-values were determined using the bootstrapping method describe in Friedman & Alm (2012), using 1000 bootstrap replicates

(Figure S9). Finally, OTU tables for each gene were initially rarefied to the lowest sequencing depth attained within the respective dataset, followed by calculation of absolute OTU abundances using qPCR data in the same manner as described for the SparCC analysis. This allowed for the proportion of OTUs to be adjusted for sequencing depth within each sample for each of the four gene targets, while avoiding the issue of compositionality. The network was then inferred using Spearman correlations (ρ), and 'RMThreshold' was again used to determine the threshold for ρ (Figure S9). For all the Spearman and Pearson based analyses, corrected P-values were determined using false-discovery rate corrections of raw P-values, and edges above 0.001 were excluded in each of the three network inferences. The resulting Pearson + RMT, SparCC and rarefied Spearman + RMT networks were then plotted in Cytoscape (v3.6) and groupings of OTUs from different lineages within each of the four functional groups (AOA, AOB, *Nitrospira* NOB and *Nitrobacter* NOB) were compared to the original Pearson-based network inference. The degree of network overlap between each inference was determined by Jaccard similarities, defined as the intersection of shared edges divided by the union of all edges in both networks. Network modularity and assortativity were also compared across the original and additional network inferences to determine the stability of these metrics. The significance of Jaccard, modularity and assortativity scores were determined using permutational tests in which network edges were randomized using the 'rewire' function in the 'igraph' package, while keeping the same degree distribution. Additionally, the connectivity of each node relative to module membership was determined by calculating its within-module connectivity (*Zi*) and participation coefficient (*Pi*) (Guimerà & Amaral 2005; Deng *et al.* 2012), which were used to classify nodes as being module hubs (highly connected within a module, $Zi > 2.5$, $Pi < 0.62$), network hubs (highly connected within the entire network, $Zi > 2.5$, $Pi > 0.62$), module connectors (nodes connecting modules, $Zi < 2.5$, $Pi > 0.62$) or peripherial (nodes connected within modules with few outside connections, $Zi < 2.5$, $Pi < 0.62$). The number of peripheral, module connector, module hub, and network hub nodes were then compared across all networks (Table S3).

Supplementary Results

Comparison of different network inferences

Based on comparison of the networks produced using the different methodologies, we found that the patterns observed in the original network (Figure 2), inferred using Pearson correlations of *rlog* transformed values, were robust. Co-occurrences between various lineages of AOA, AOB, *Nitrospira* and *Nitrobacter* were largely similar across all the networks (Figures S7, S9 and S10), which was confirmed by significant Jaccard similarities. However, some differences were also observed between each of the networks, with no two methods providing the exact same network structure.

The use of random matrix theory to define a threshold for the original Pearson adjacency matrix based on *rlog*-transformed showed that modules observed in the original network (Figure 2) were largely retained, though module 4 in the original network was divided into two adjacent modules, 5 and 8, in the Pearson + RMT network (Figure S7). We observed a range of candidate thresholds based on the 'RMThreshold output' (Figure S8) and a value of 0.71 was selected as being the most likely point at which the nearest neighbor spacing distribution transitions to a Poisson (exponential) distribution. However, lower values, including the original threshold of 0.64, were also potential thresholds. The resulting network was significantly similar to the original inference using $r \geq 0.64$ as a cutoff value, and node degree distribution also followed the power law indicating a scale-free topology. Modularity and assortativity scores were similar to those of the original network (Table S3).

The SparCC method, when used with absolute abundances, resulted in the most complex network (Figure S9). The P-value cutoff was 0.001, as recommended in previous studies (Weiss *et al.*, 2016). When setting the correlation threshold at 0.3, the number of retained nodes and edges did not decrease appreciably (650 to 642 nodes, 5287 to 5238 edges). However, visual inspection showed a similar segregation of various lineages within the four functional groups into different modules compared the original inference, with a significant Jaccard similarity of 0.23 ($P < 0.001$; Table S4). Similarity to the Pearson/RMT network was lower yet still significant (0.15, $P < 0.001$), likely due to the greater number of edges observed in the SparCC network. A notable difference observed in the SparCC network was an increased presence and stronger association of *Nitrobacter nxrB* OTUs from the *N. vulgaris* subgroup 4.2 lineage with one of the main modules (module 1) in the network. Many of the same OTUs were also observed in the original network as module 12 (Figure 2), but were marginally connected to other modules in the network. Node degree distribution again indicated a scale-free topology, and modularity and assortativity scores of the SparCC network were lower than those determined for the original network, yet still significant (Table S3).

The combined use of RMT thresholding with Spearman correlations of the rarefied absolute abundance table also showed a more prominent appearance of the *N. vulgaris* subgroup 4.2 *Nitrobacter nxrB* lineage in the network (Figure S10), although these OTUs formed their own module that was marginally associated with the other large modules in the network, similar to the original network inference. A range of candidate threshold values was again observed, and we selected a value of 0.61 as being the most likely threshold for identifying non-random patterns in the network. Similarity to the original network inference was confirmed by a significant Jaccard similarity, and the distribution of node degree again indicated a scale-free topology (Table S3). Modularity of the rarefied Spearman network was comparable to that of the original inference, while assortativity was slightly lower, yet still significant (Table S3). The rarefied/Spearman and SparCC were also significantly similar (0.18, $P > 0.001$), however similarities to the Pearson + RMT (0.26, $P < 0.001$) and the original network were higher.

Node connectivity and module membership

Classification of nodes as peripheral, module hubs, module connectors and network connectors showed that the proportion of nodes classified into the different categories were largely similar across the different networks (Table S3), although the *rlog*/Pearson/RMT inference had few module hub and connector nodes. However, the identification of these nodes fluctuated across the inferences; seven nodes were commonly classified as module hubs and connecters between the original and SparCC networks, whereas only two nodes were classified as module connectors in both the original and the rarefied Spearman networks. Despite these differences, a similar pattern of a higher number of module connector nodes than module hub nodes was observed across the different inferences, suggesting a general ecological pattern that is not an artifact of network inference.

Table S1. Primers and thermal cycling conditions for amplification of *amoA* genes from ammonia oxidizing archaea and bacteria (AOA and AOB, respectively), and *nxrB* genes from *Nitrospira* and *Nitrobacter* type nitrite oxidizers for sequencing on 454 or Illumina MiSeq platforms, as well as quantitative real-time PCR conditions.

**Group (platform)**	**Primer Name**	**Sequence**	**Amplicon Size**	**PCR conditions***	**Reference**
AOA (454)	CamoA-19f	ATG GTC TGG YTW AGA CG	629	98°C 3 min; (98°C 30 sec, 50°C 60 sec, 72°C 60 sec) × 25; 72°C 5 min	Pester et al., 2012
	CamoA-616r	GCC ATC CAB CKR TAN GTC CA			
AOB (MiSeq)	amoA-1F	GGG GTT TCT ACT GGT GGT	492	98°C 3 min; (98°C 30 sec, 55°C 30 sec, 72°C 45 sec) × 30; 72°C 10 min	Rotthauwe et al., 1997
	amoA-1R	CCC CTC KGS AAA GCC TTC TTC			
Nitrospira NOB (MiSeq)	nxrB169f	TAC ATG TGG TGG AAC A	485	98°C 3 min; (98°C 30 sec, 56°C 30 sec, 72°C 45 sec) × 30; 72°C 10 min	Pester et al., 2013
	nxrB638r	CGG TTC TGG TCR ATC A			
Nitrobacter NOB (MiSeq)	nxrB1F	ACG TGG AGA CCA AGC CGG G	380	98°C 3 min; (98°C 30 sec, 66°C 30 sec, 72°C 45 sec) × 33; 72°C 10 min	Vanparys et al., 2007
	nxrB1R	CCG TGC TGT TGA YCT CGT TGA			
Nitrospira NOB (qPCR)	Same primer set as used for sequencing		492	95°C 3 min; (95°C 30 sec, 56°C 30 sec, 72°C 40 sec; 78°C 10 sec) × 35; 72°C 10 min; melt curve 72°C-95°C, 0.5°C increments	
Nitrobacter NOB (MiSeq)	Same primer set as used for sequencing		380	95°C 3 min; (95°C 30 sec, 72 – 67 °C 30 sec, 72°C 30 sec) × 8; (95°C 30 sec, 67 °C 30 sec, 72°C 40 sec) × 35 72°C 10 min; melt curve 72°C-95°C, 0.5°C increments	

*Conditions for 454 and MiSeq sequencing amplification describe the initial PCR of target amplicons from environmental samples.

Table S2. Sequencing results and number of OTUs for AOA, AOB, *Nitrospira* and *Nitrobacter* NOB datasets.

Group	Number of raw reads after merging[*]	Number of reads after quality filtering	Percent of filtered reads retained after chimera removal and OTU analysis	Number of OTUs (97%, no singletons)
AOA	264402	224383	99.1%	162
AOB	4857157	4845286	94.3%	98
Nitrobacter NOB	8663632	8371726	99.6%	115
Nitrospira NOB	4744672	4702223	88.2%	653

[*]Does not apply to 454 data.

Table S3. Comparison of network properties across networks structure inferred using Pearson correlations of regularized log (*rlog*) transformed OTU abundances, random matrix theory (RMT) defined threshold values, SparCC and Spearman correlations of rarefied total OTU abundances.

Network properties	Network inference method			
	rlog Pearson[a]	*rlog* Pearson + RMT[b]	SparCC	Rarefied Spearman + RMT
Minimum correlation	$r \geq 0.64$	$r \geq 0.71$	$r \geq 0.18$	$\rho \geq 0.61$
Nodes	520	380	650	483
Edges	2293	1063	5287	1415
Jaccard similarity to original network (score, *P*)[b]	-	0.46 (P < 0.001)	0.24 (P < 0.001)	0.25 (P < 0.001)
Modularity (score, *P*)[b]	0.68 (P<0.001)	0.74 (P < 0.001)	0.59 (P < 0.001)	0.71 (P < 0.001)
Assortativity (score, *P*)[b]	0.19 (P<0.001)	0.19 (P < 0.001)	0.11 (P < 0.001)	0.13 (P < 0.001)
Power-law fit (α in $x^{-\alpha}$, *P*)[c]	2.0 (P = 0.85)	1.96 (P = 0.98)	2.28 (P = 0.76)	1.86 (P = 0.97)
Peripheral nodes	505	297	596	471
Module Connectors	14	2	49	11
Module hubs	1	1	5	1
Network hubs	0	0	3	0

[a]Original network, threshold based on fit of node degree distribution to power law.
[b]*P*-values based on permutation tests, *n*=1000
[c]Non-significant *P*-values indicate adequate fit of node degree distribution to power law based on Kolmorgorov-Smirnov test.

Table S4. Moran's *I* tests, variogram parameters and results from cross-validation estimation of prediction errors for geostatistical analysis of *Nitrospira* and *Nitrobacter nxrB* gene abundance, as well as modules of co-occurring ammonia oxidizing/nitrite oxidizing communities with significant spatial autocorrelation prior to interpolation

	**Moran's *I* (Z-score; P-value)**	**Model**	**C_0+C (sill, $unit^2$)**	**R (range, m)**	**C_0 (nugget, $unit^2$)**	**r^a**	**$RMSEP^b$**	**RPD^c**
Nitrospira nxrB copies g^{-1} soil DW*	2.06; 0.035	Spherical	0.0316	234	0.0008	0.396	0.154	1.07
Nitrobacter nxrB copies g^{-1} soil DW	1.98; 0.041	Exponential	1.01×10^5	325	4.63×10^3	0.452	229.06	1.12
Nitrospira nxrB:*Nitrobacter nxrB*	3.88; 0.004	Spherical	0.369	341	0.117	0.588	0.516	1.25
Module 1†	5.31; 0.001	Exponential	0.081	158	0.013	0.623	0.226	1.29
Module 2†	3.77; 0.004	Circular	0.076	189	0.017	0.394	0.269	1.08
Module 3†	3.93; 0.004	Exponential	0.074	100	0.012	0.543	0.243	1.20
Module 4†	1.96; 0.043	Circular	0.091	164	0.002	0.4	0.268	1.09
Module 8†	5.54; 0.002	Circular	0.061	410	0.031	0.581	0.236	1.24
Module 9†	6.06; 0.001	Circular	0.07	129	0.0004	0.525	0.246	1.19

[a]Pearson's correlation between observed and predicted values based on model fit
[b]Root mean squared error of prediction
[c]Ratio of performance deviation, the standard deviation of the variable divided by RMSEP
*Values $\log_{10}$ transformed prior to variogram fitting
†Values rank transformed prior to variogram fitting

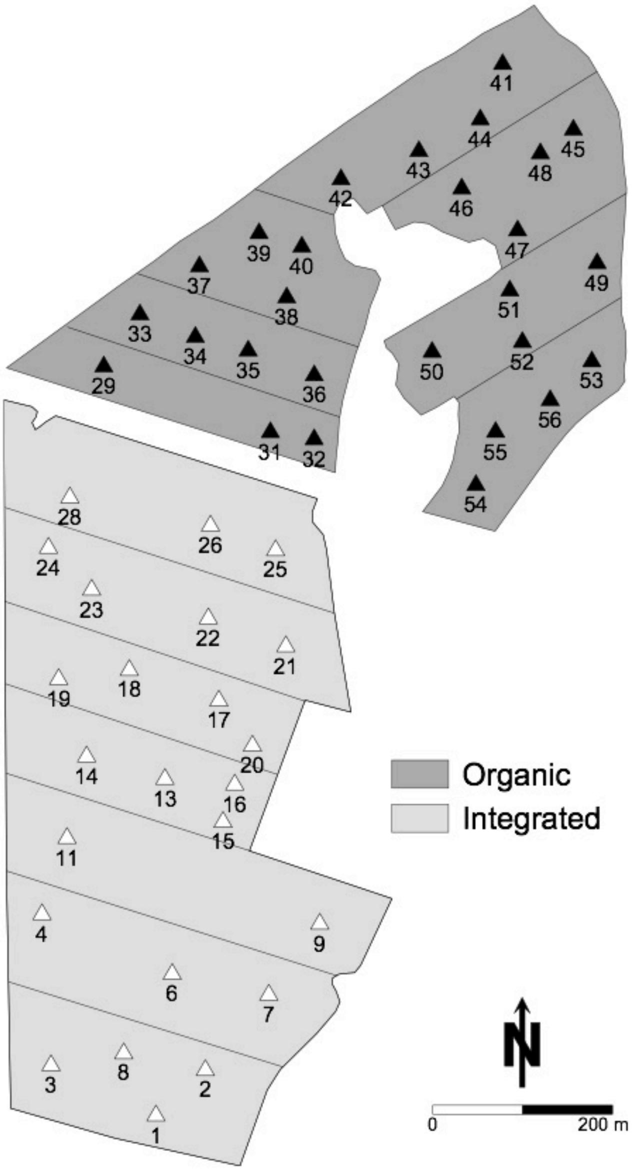

Figure S1. The Logården experimental farm (44 ha). This farm is divided into seven fields in the southern area (26 ha, light gray) under integrated crop management, and seven fields in the northern area (18 ha, dark gray) under organic management. Sampling points are indicated.

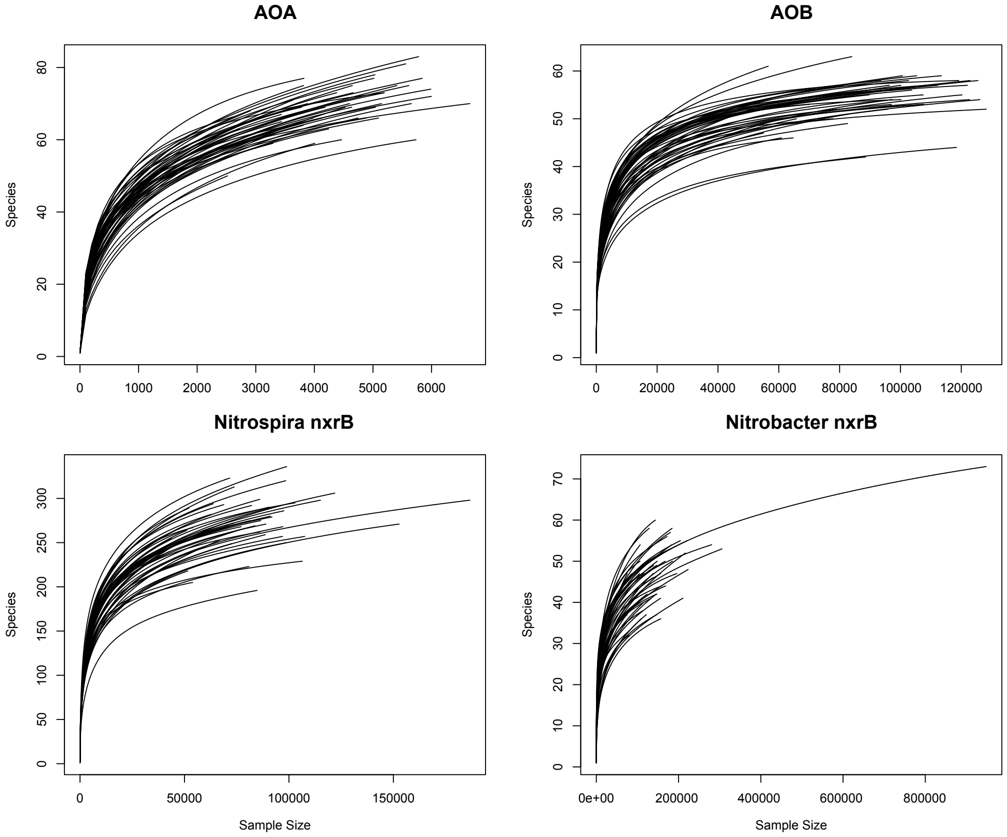

Figure S2. Rarefaction curves of ammonia oxidizing archaea (AOA), bacteria (AOB) and both *Nitrospira* and *Nitrobacter* nitrite oxidizing communities for all sampling points.

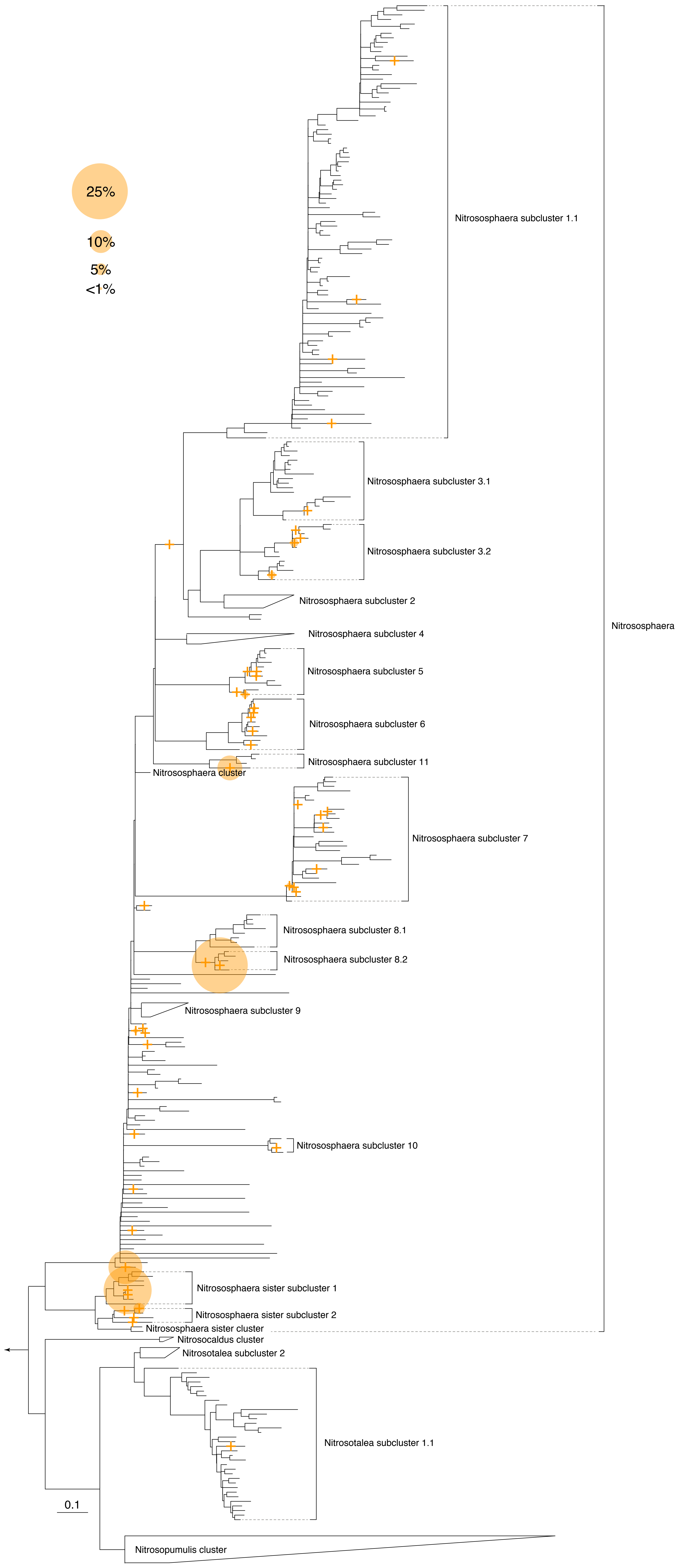

Figure S3. Phylogenetic placement of ammonia oxidizing archaeal (AOA) OTUs within the reference phylogeny of AOA amoA sequences determined by Pester et al. (2012), with the different lineages described therein indicated. Crosses show location of mapped OTUs within the reference tree, and circle size is proportional the relative abundance of reads mapping to a particular lineage. Scale bar denotes estimated nucleotide substitution rate. Tips names have been excluded for clarity.

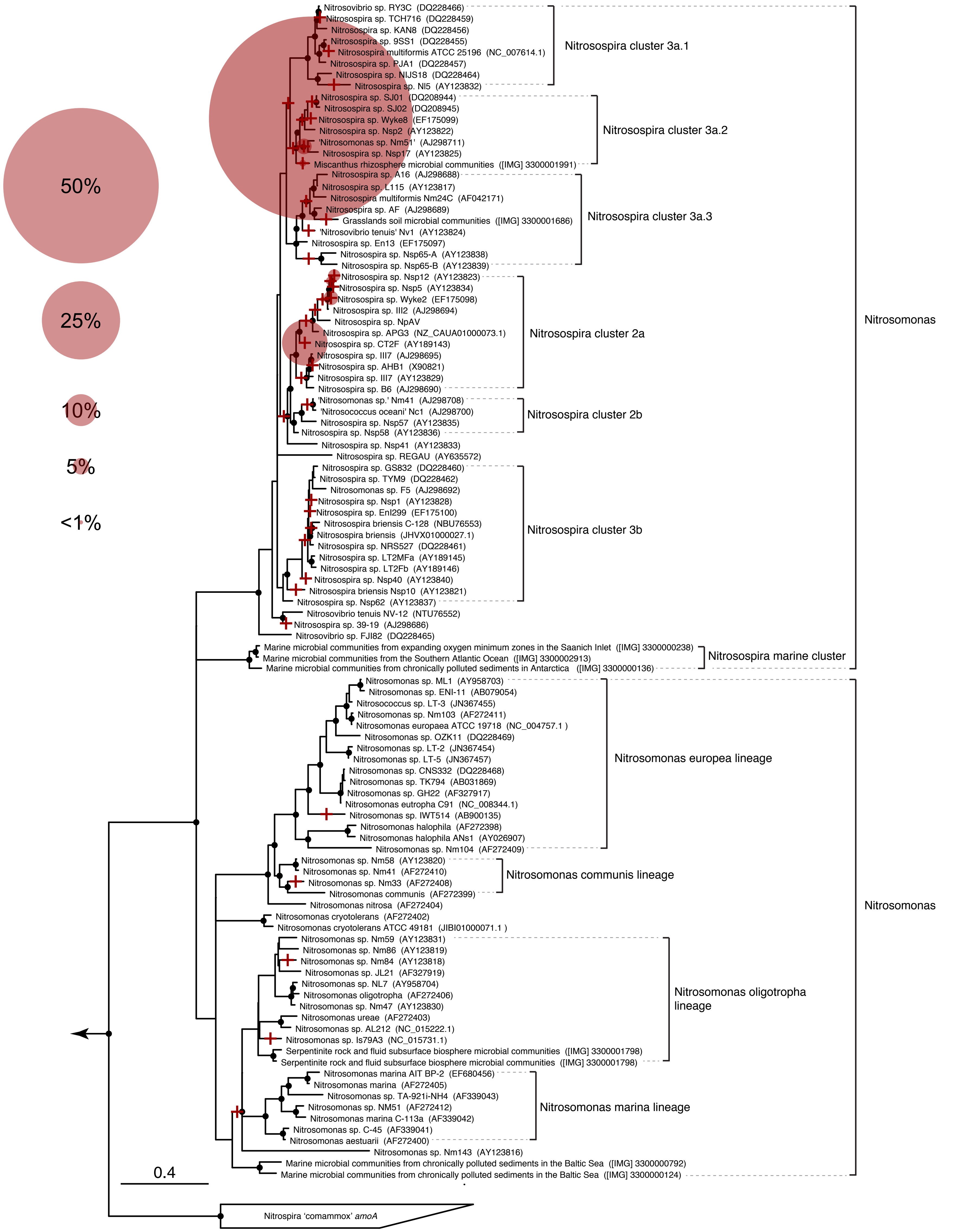

Figure S4. Phylogenetic placement of ammonia oxidizing bacterial (AOB) OTUs within the reference nucleotide phylogeny (this study, ExaBayes GTR+Γ) of AOB amoA sequences obtained from pure culture studies and metagenomes. Lineages described in previous studies are shown at right, and crosses show location of mapped OTUs within the reference tree. Circle size is proportional the relative abundance of reads mapping to a particular lineage, and scale bar denotes estimated nucleotide substitution rate. Clades with >90% Bayesian posterior probabilities are indicated by (●).

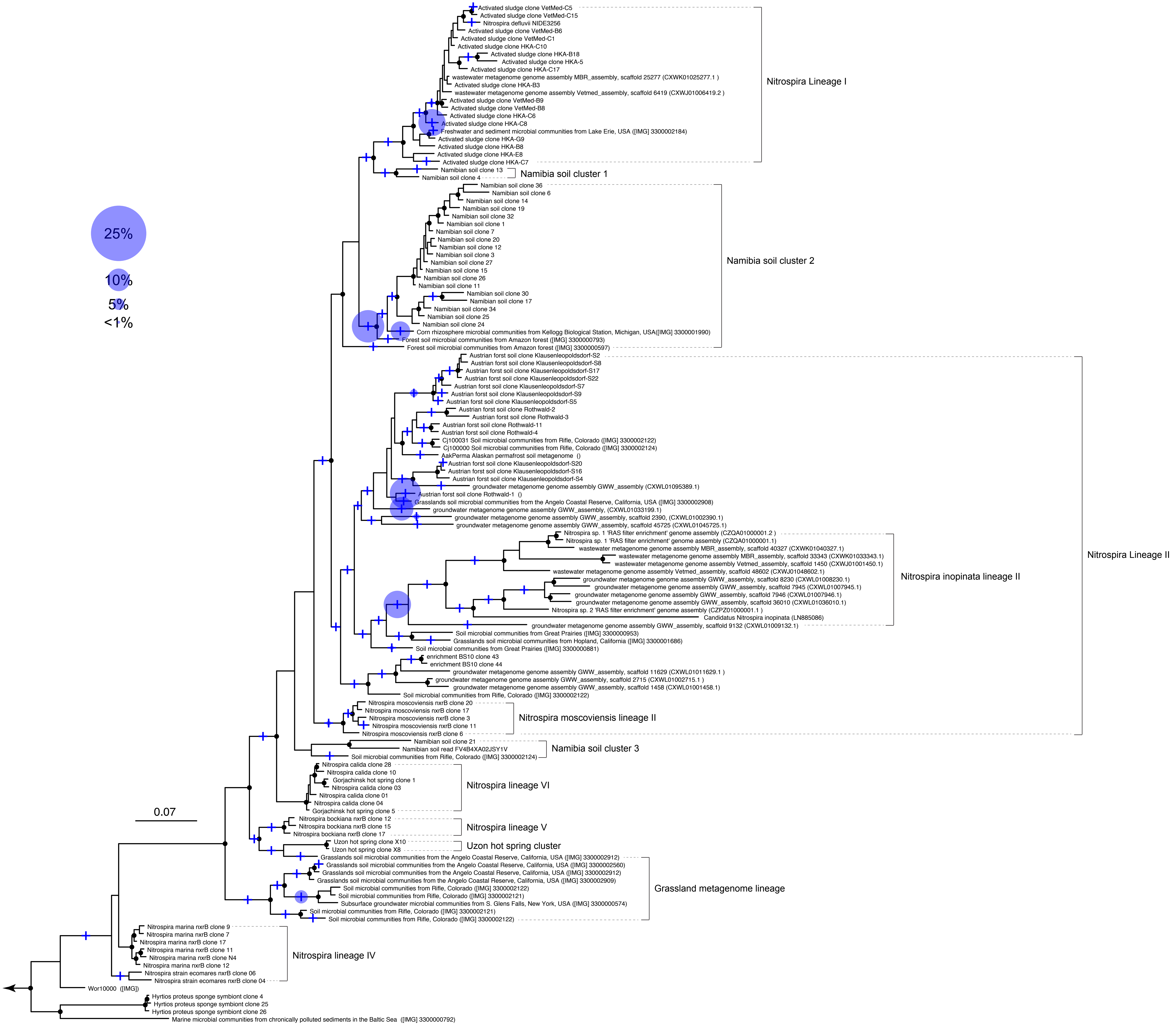

Figure S5. Phylogenetic placement of Nitrospira OTUs within the reference nucleotide phylogeny (this study, IQTREE maximum likelihood GTR+Γ) of Nitrospira nxrB sequences obtained from Pester et al. (2013), as well as assembled metagenomes. Lineages described in Pester et al. (2013) are shown at right, and crosses show location of mapped OTUs within the reference tree. Circle size is proportional the relative abundance of reads mapping to a particular lineage, and scale bar denotes estimated nucleotide substitution rate. Clades with >80% ultrafast bootstrap probabilities are indicated by (●).

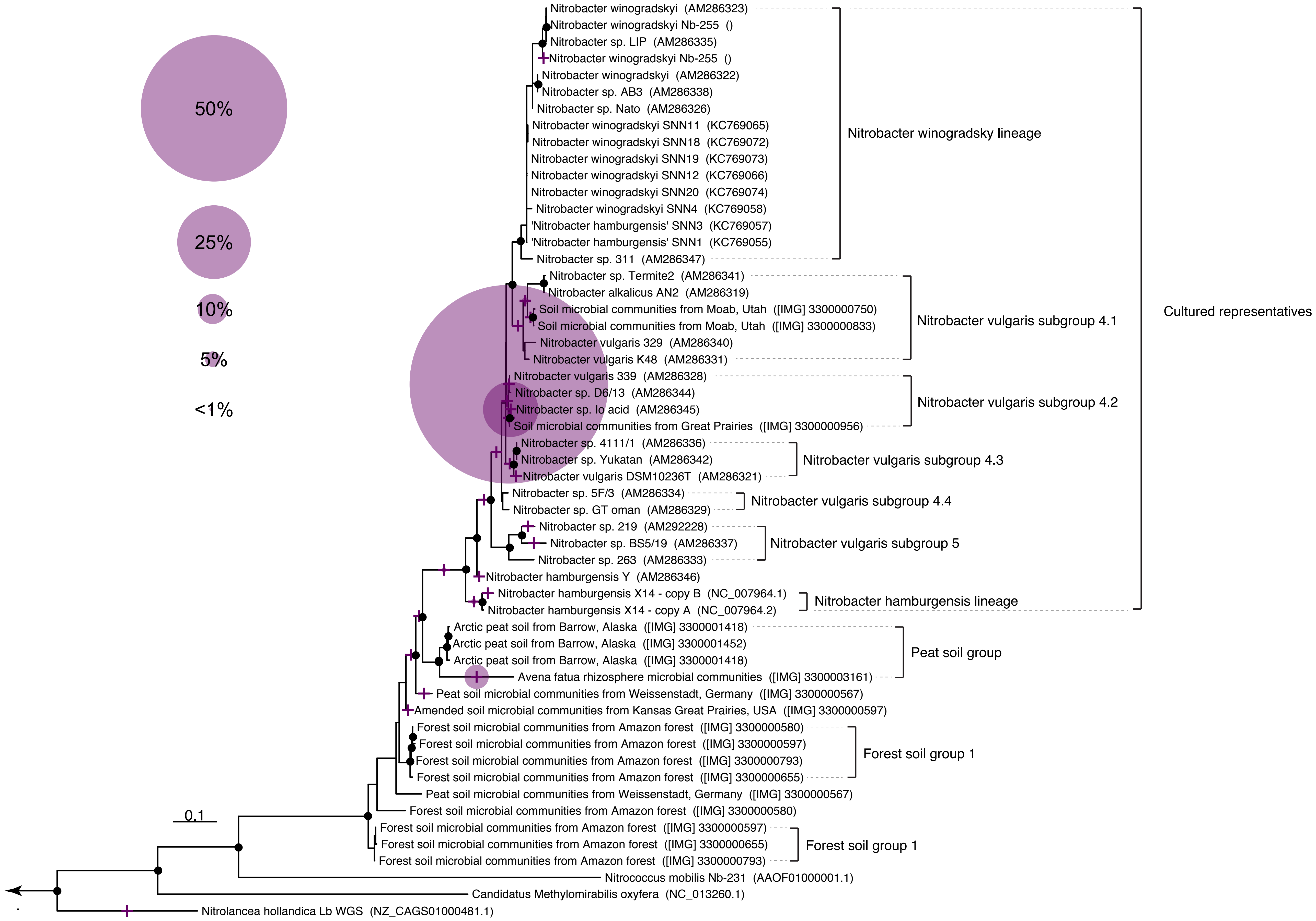

Figure S6. Phylogenetic placement of Nitrobacter OTUs within the reference nucleotide phylogeny (this study, ExaBayes GTR+Γ) of Nitrobacter nxrB sequences obtained from pure culture studies as well as assembled metagenomes. Lineages described in Vanparys et al. (2007) are shown at right, and crosses show location of mapped OTUs within the reference tree. Circle size is proportional the relative abundance of reads mapping to a particular lineage, and scale bar denotes estimated nucleotide substitution rate. Clades with >90% Bayesian posterior probabilities are indicated by (●).

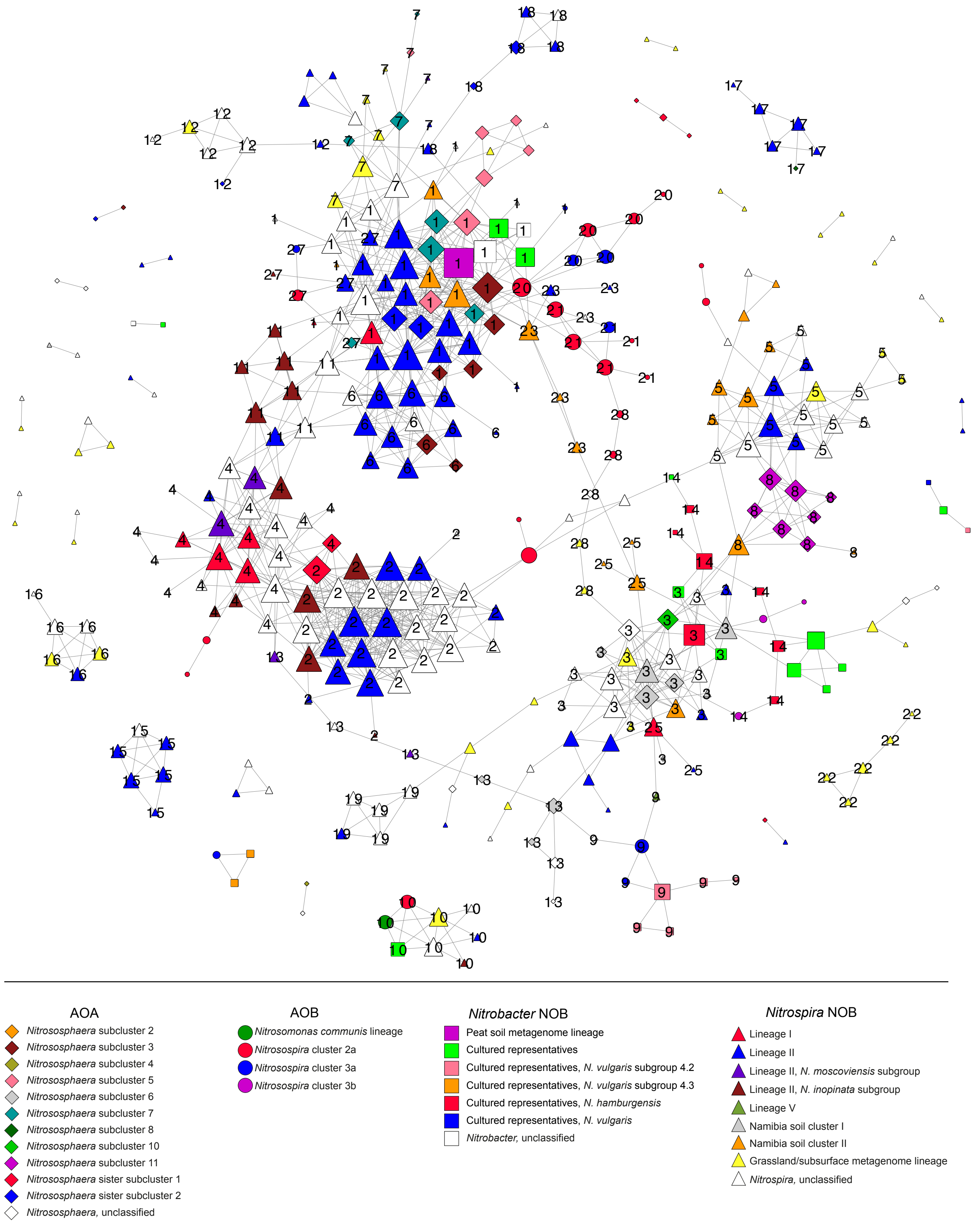

Figure S7. Network analysis of co-occurring AOA, AOB, *Nitrospira* and *Nitrobacter* OTUs based on Pearson correlations (*r*) of regularized log transformed OTU abundances. Node size is proportional to node degree, while connections between each node indicate significant positive correlations above the set threshold as determined by random matrix theory ($P < 0.001$, $r \geq 0.71$). The shape and color of the nodes denote the functional group and lineage, respectively, of each OTU, while the numbers indicate membership of co-occurring OTUs to distinct community modules. Nodes with degree = 0 are not shown.

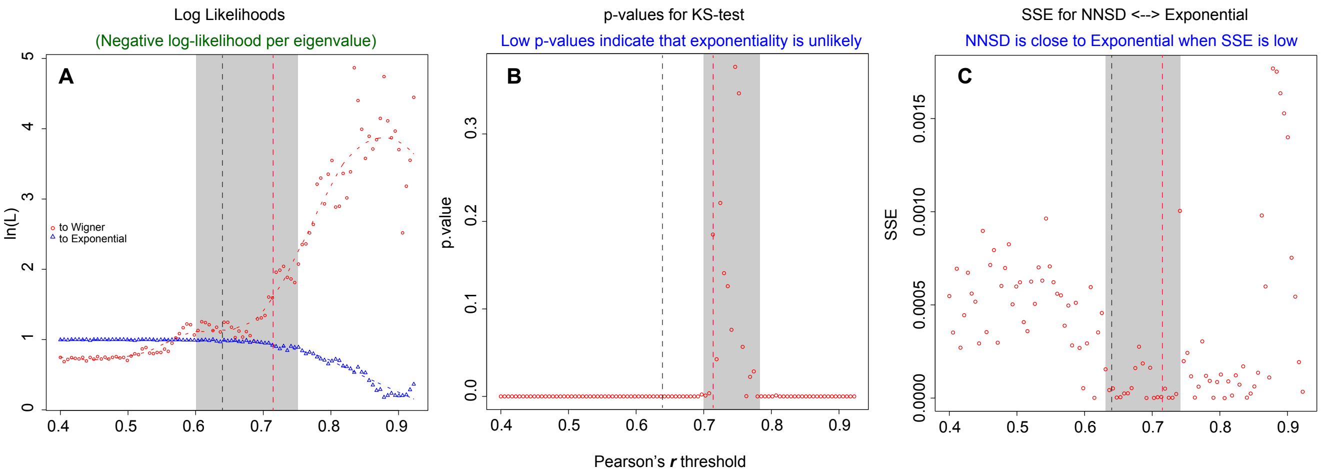

Figure S8. Results from RMThreshold used to determine threshold value of Pearson's *r* (dotted vertical red line) for the regularized log transformed abundnaces. The grey shaded area indicates regions of candidate threshold values considered in this analysis, while the black dotted line shows the threshold value determined in the original analysis based on fit of degree distribution to power law. A) Log-likelihood plot of distances between nearest neighbor spacing distribution (NNSD) and both Gaussion orthonormal ensemble ('Wigner') or Poisson distirbutions ('Exponential'). The point at which the lines begin to diverge indicate potential threshold values where the matrix displays non-random association between eigenvectors. B) Kolmogorov-Smirnov test of NNSD against the Poisson distribution. Values of ρ with a probability greater than 0.01 are considered potential candidate thresholds. C) Sum of squared errors between eimpirical NNSD and Possion distribution compared to ρ. Values close to zero indicate candidate threshold values.

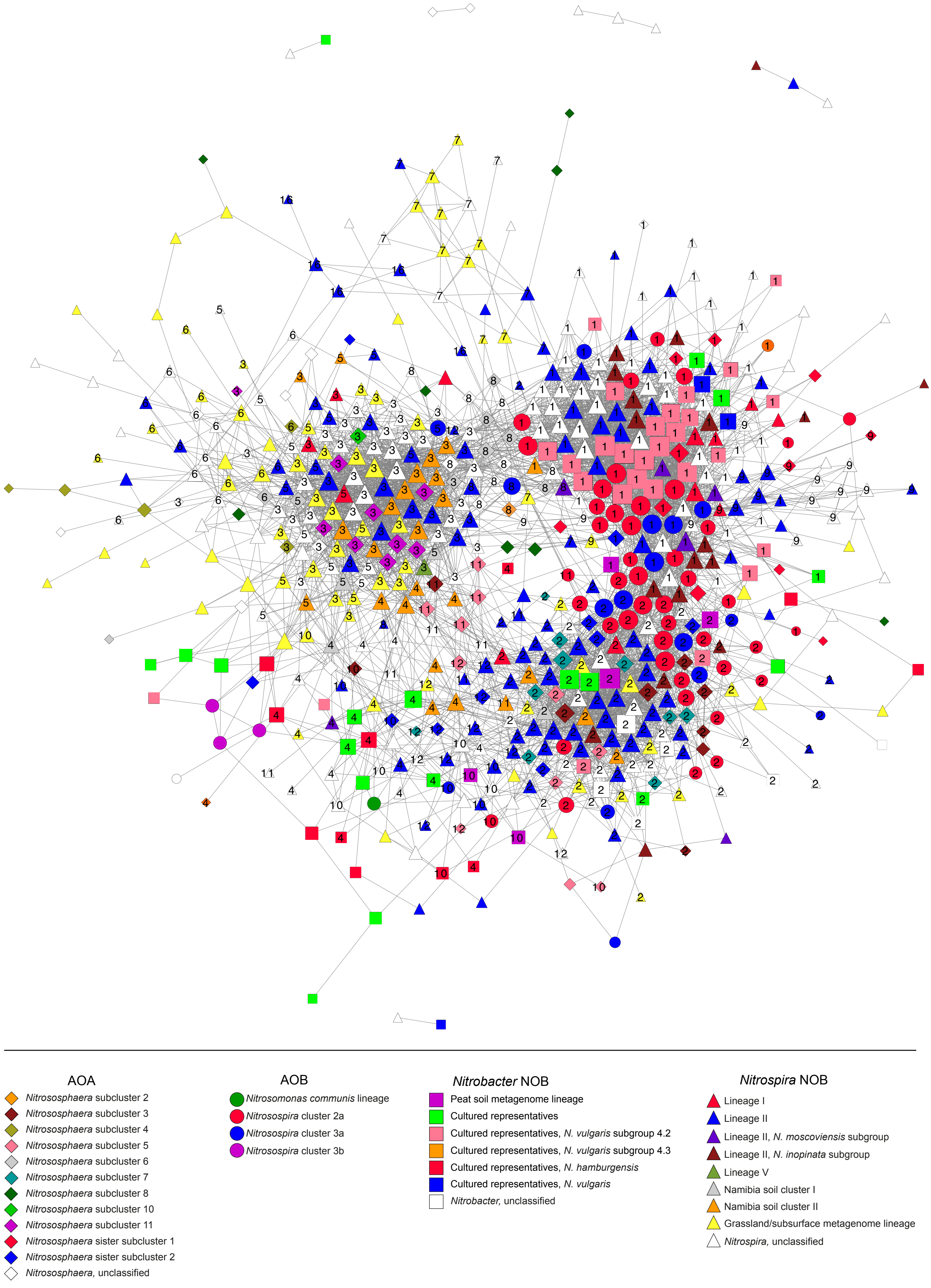

Figure S9. Network analysis of co-occurring AOA, AOB, *Nitrospira* and *Nitrobacter* OTUs based on SparCC correlations of total OTU abundances. Node size is proportional to node degree, while connections between each node indicate significant positive correlations ranging from 0.18 to 1 (pseudo P-value < 0.001, 1000 bootstrap samples). The shape and color of the nodes denote the functional group and lineage, respectively, of each OTU, while the numbers indicate membership of co-occurring OTUs to distinct community modules. Nodes with degree = 0 are not shown.

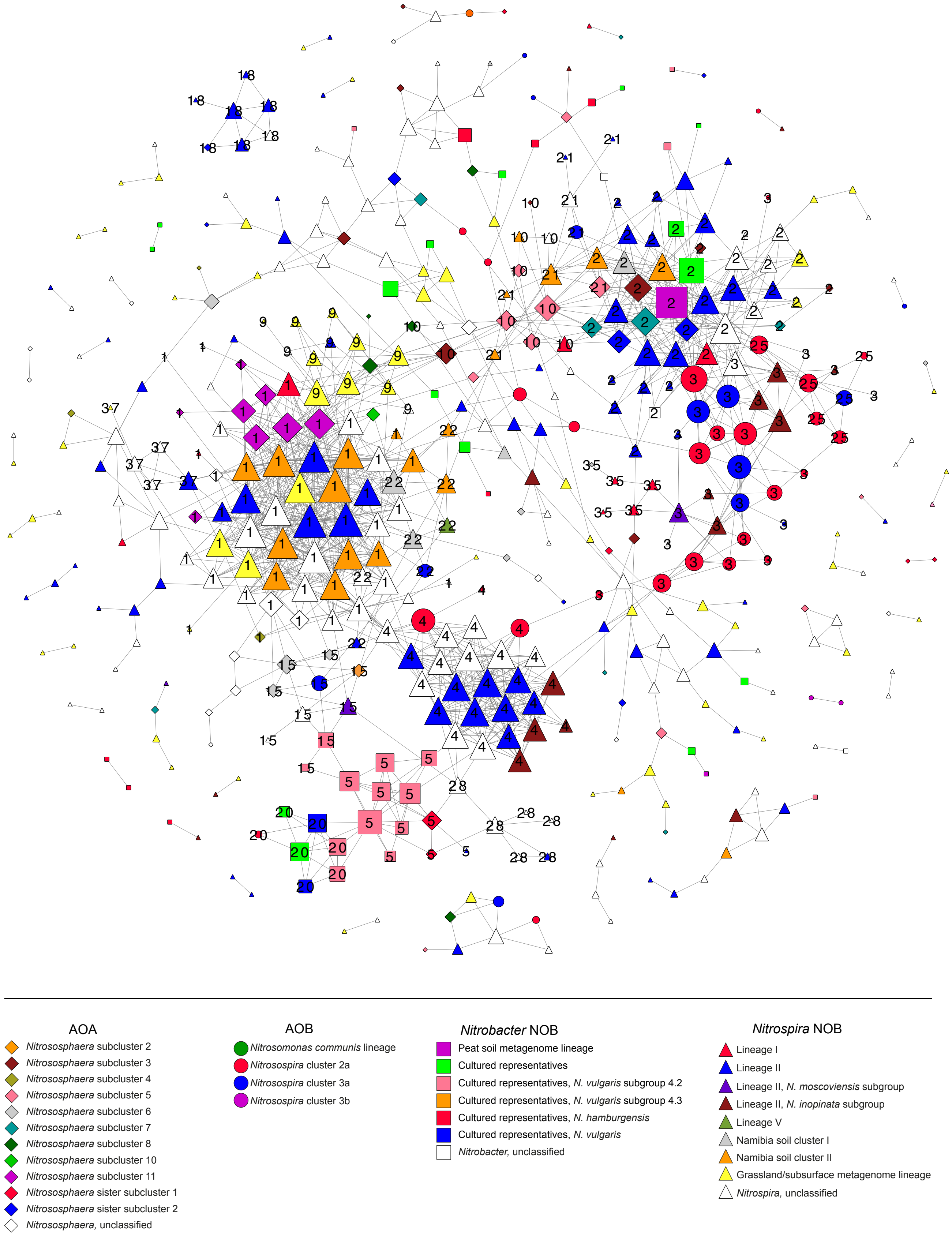

Figure S10. Network analysis of co-occurring AOA, AOB, *Nitrospira* and *Nitrobacter* OTUs based on Spearman correlations (ρ) of rarefied total OTU abundances. Node size is proportional to node degree, while connections between each node indicate significant positive correlations above the set threshold as determined by random matrix theory ($P < 0.001$, $r \geq 0.61$). The shape and color of the nodes denote the functional group and lineage, respectively, of each OTU, while the numbers indicate membership of co-occurring OTUs to distinct community modules. Nodes with degree = 0 are not shown.

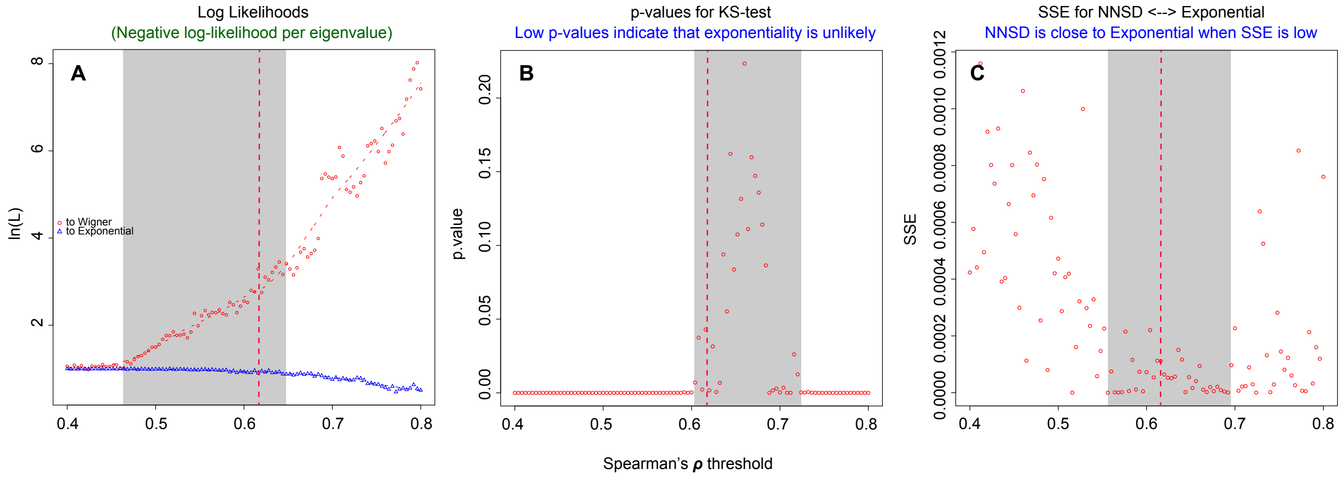

Figure S11. Results from RMThreshold used to determine threshold value of Spearman's ρ (dotted vertical red line) for the rarefied, total abundance network. The grey shaded area indicates regions of candidate threshold values considered in this analysis. A) Log-likelihood plot of distances between nearest neighbor spacing distribution (NNSD) and both Gaussion orthonormal ensemble ('Wigner') or Poisson distirbutions ('Exponential'). The point at which the lines begin to diverge indicate potential threshold values where the matrix displays non-random association between eigenvectors. B) Kolmogorov-Smirnov test of NNSD against the Poisson distribution. Values of ρ with a probability greater than 0.01 are considered potential candidate thresholds. C) Sum of squared errors between eimpirical NNSD and Possion distribution compared to ρ. Values close to zero indicate candidate threshold values.

References

Aberer AJ, Kobert K, Stamatakis A (2014) ExaBayes: massively parallel bayesian tree inference for the whole-genome era. *Molecular Biology And Evolution*, **31**, 2553–2556.

Avrahami S, Conrad R, Braker G (2002) Effect of soil ammonium concentration on N2O release and on the community structure of ammonia oxidizers and denitrifiers. *Applied And Environmental Microbiology*, **68**, 5685–5692.

Berry D, Ben Mahfoudh K, Wagner M, Loy A (2011) Barcoded primers used in multiplex amplicon pyrosequencing bias amplification. *Applied And Environmental Microbiology*, **77**, 7846–7849.

Caporaso JG, Kuczynski J, Stombaugh J *et al.* (2010) QIIME allows analysis of high-throughput community sequencing data. *Nature Methods*, **7**, 335–336.

Daims H, Lebedeva EV, Pjevac P *et al.* (2015) Complete nitrification by *Nitrospira* bacteria. *Nature*, **528**, 504–509.

Deng Y, Jiang Y-H, Yang Y *et al.* (2012) Molecular ecological network analyses. *BMC bioinformatics*, **13**, 113.

Eddy S (1998) Profile hidden Markov models. *Bioinformatics*, **14**, 755–763.

Edgar RC (2010) Search and clustering orders of magnitude faster than BLAST. *Bioinformatics*, **26**, 2460–2461.

Edgar RC (2013) UPARSE: highly accurate OTU sequences from microbial amplicon reads. *Nature Methods*, **10**, 996–998.

Friedman J, Alm EJ (2012) Inferring correlation networks from genomic survey data. (C von Mering, Ed,). *PLoS Computational Biology*, **8**, e1002687.

Gruber-Dorninger C, Pester M, Kitzinger K *et al.* (2015) Functionally relevant diversity of closely related *Nitrospira* in activated sludge. *The ISME Journal*, **9**, 643–655.

Guimerà R, Amaral LAN (2005) Functional cartography of complex metabolic networks. *Nature*, **433**, 895–900.

Hoang DT, Chernomor O, Haeseler von A, Minh BQ, Le Sy Vinh (2018) UFBoot2: Improving the Ultrafast Bootstrap Approximation. *Molecular Biology and Evolution*, **35**, 518–522.

Katoh K, Standley DM (2013) MAFFT Multiple Sequence Alignment Software Version 7: Improvements in Performance and Usability. *Molecular Biology And Evolution*, **30**, 772–780.

Kunin V, Engelbrektson A, Ochman H, Hugenholtz P (2010) Wrinkles in the rare biosphere: pyrosequencing errors can lead to artificial inflation of diversity estimates. *Environmental Microbiology*, 118–123.

Ludwig W, Strunk O, Westram R *et al.* (2004) ARB: a software environment for sequence data. *Nucleic Acids Research*, **32**, 1363–1371.

Markowitz VM, Chen I-MA, Palaniappan K *et al.* (2012) IMG: the integrated microbial genomes database and comparative analysis system. *Nucleic Acids Research*, **40**, D115–D122.

Menzel U (2016) RMThreshold: signal-noise separation in random matrices by using eigenvalue spectrum analysis. R package version 1.1. https://CRAN.R-project.org/package=RMThreshold

Mintie AT, Heichen RS, Cromack K, Myrold DD, Bottomley PJ (2003) Ammonia-oxidizing bacteria along meadow-to-forest transects in the Oregon Cascade Mountains. *Applied And Environmental Microbiology*, **69**, 3129–3136.

Nguyen L-T, Schmidt HA, Haeseler von A, Minh BQ (2015) IQ-TREE: A Fast and Effective Stochastic Algorithm for Estimating Maximum-Likelihood Phylogenies. *Molecular Biology and Evolution*, **32**, 268–274.

Pester M, Maixner F, Berry D *et al.* (2013) NxrB encoding the beta subunit of nitrite oxidoreductase as functional and phylogenetic marker for nitrite‐oxidizing Nitrospira. *Environmental Microbiology*, **16**, 3055–3071.

Pester M, Rattei T, Flechl S *et al.* (2012) *amoA*-based consensus phylogeny of ammonia-oxidizing archaea and deep sequencing of *amoA* genes from soils of four different geographic regions. *Environmental Microbiology*, **14**, 525–539.

Purkhold U, Pommerening-Roser A, Juretschko S *et al.* (2000) Phylogeny of all recognized species of ammonia oxidizers based on comparative 16S rRNA and amoA sequence analysis: implications for molecular diversity surveys. *Applied And Environmental Microbiology*, **66**, 5368–5382.

Purkhold U, Wagner M, Timmermann G, Pommerening-Röser A, Koops H-P (2003) 16S rRNA and amoA-based phylogeny of 12 novel betaproteobacterial ammonia-oxidizing isolates: extension of the dataset and proposal of a new lineage within the nitrosomonads. *International Journal Of Systematic Bacteriology*, **53**, 1485–1494.

Rotthauwe J, Witzel K (1997) The ammonia monooxygenase structural gene amoA as a functional marker: molecular fine-scale analysis of natural ammonia-oxidizing populations. *Applied And Environmental Microbiology*, **63**, 4704–4712.

Schloss PD, Westcott SL, Ryabin T *et al.* (2009) Introducing mothur: open-source, platform-independent, community-supported software for describing and comparing microbial communities. *Applied And Environmental Microbiology*, **75**, 7537–7541.

van Kessel MAHJ, Speth DR, Albertsen M *et al.* (2015) Complete nitrification by a single microorganism. *Nature*, **528**, 555–559.

Vanparys B, Spieck E, Heylen K *et al.* (2007) The phylogeny of the genus *Nitrobacter* based

on comparative rep-PCR, 16S rRNA and nitrite oxidoreductase gene sequence analysis. *Systematic and Applied Microbiology*, **30**, 297–308.

Waddell P, Steel M (1997) General time-reversible distances with unequal rates across sites: Mixing Gamma and inverse gaussian distributions with invariant sites. *Molecular Phylogenetics and Evolution*, **8**, 398–414.

Wang Q, Garrity G, Tiedje J, Cole J (2007) Naive Bayesian classifier for rapid assignment of rRNA sequences into the new bacterial taxonomy. *Applied And Environmental Microbiology*, **73**, 5261–5267.

Weiss S, Van Treuren W, Lozupone C *et al.* (2016) Correlation detection strategies in microbial data sets vary widely in sensitivity and precision. *The ISME Journal*, **10**, 1669–1681.

Zhang J, Kobert K, Flouri T, Stamatakis A (2014) PEAR: a fast and accurate Illumina Paired-End reAd mergeR. *Bioinformatics*, **30**, 614–620.

Zhang Y, Sun Y (2011) HMM-FRAME: accurate protein domain classification for metagenomic sequences containing frameshift errors. *BMC bioinformatics*, **12**, 198.
